# Microglia mediate neurocognitive deficits by eliminating C1q-tagged synapses in sepsis-associated encephalopathy

**DOI:** 10.1126/sciadv.abq7806

**Published:** 2023-05-26

**Authors:** Ha-Yeun Chung, Jonathan Wickel, Nina Hahn, Nils Mein, Meike Schwarzbrunn, Philipp Koch, Mihai Ceanga, Holger Haselmann, Carolin Baade-Büttner, Nikolai von Stackelberg, Nina Hempel, Lars Schmidl, Marco Groth, Nico Andreas, Juliane Götze, Sina M. Coldewey, Michael Bauer, Christian Mawrin, Justina Dargvainiene, Frank Leypoldt, Stephan Steinke, Zhao-Qi Wang, Michael Hust, Christian Geis

**Affiliations:** ^1^ Section of Translational Neuroimmunology, Department of Neurology, Jena University Hospital, Jena 07747, Germany.; ^2^ Center for Sepsis Control and Care, Jena University Hospital, Jena 07747, Germany.; ^3^ Leibniz Institute on Aging - Fritz Lipmann Institute (FLI), Jena 07745, Germany.; ^4^ Institute of Immunology, Jena University Hospital, Jena 07743, Germany.; ^5^ Department of Anaesthesiology and Intensive Care Medicine, Jena University Hospital, Jena 07747, Germany.; ^6^ Septomics Research Center, Jena University Hospital, Jena 07745, Germany.; ^7^ Department of Neuropathology, University of Magdeburg, Magdeburg, Germany.; ^8^ Section of Pathology, Institute of Forensic Medicine, Jena University Hospital, Jena 07749, Germany.; ^9^ Neuroimmunology, Institute of Clinical Chemistry, UKSH, Kiel/Lübeck, Germany.; ^10^ Department of Neurology, Christian-Albrechts University, Kiel 24105, Germany.; ^11^ Department Medical Biotechnology, Institute of Biochemistry, Biotechnology and Bioinformatics, Technische Universität Braunschweig, Braunschweig, Germany.; ^12^ Faculty of Biological Sciences, Friedrich Schiller University, Jena 07745, Germany.; ^13^ State Key Laboratory of Microbial Technology, Shandong University, Qingdao, China.

## Abstract

Sepsis-associated encephalopathy (SAE) is a severe and frequent complication of sepsis causing delirium, coma, and long-term cognitive dysfunction. We identified microglia and C1q complement activation in hippocampal autopsy tissue of patients with sepsis and increased C1q-mediated synaptic pruning in a murine polymicrobial sepsis model. Unbiased transcriptomics of hippocampal tissue and isolated microglia derived from septic mice revealed an involvement of the innate immune system, complement activation, and up-regulation of lysosomal pathways during SAE in parallel to neuronal and synaptic damage. Microglial engulfment of C1q-tagged synapses could be prevented by stereotactic intrahippocampal injection of a specific C1q-blocking antibody. Pharmacologically targeting microglia by PLX5622, a CSF1-R inhibitor, reduced C1q levels and the number of C1q-tagged synapses, protected from neuronal damage and synapse loss, and improved neurocognitive outcome. Thus, we identified complement-dependent synaptic pruning by microglia as a crucial pathomechanism for the development of neuronal defects during SAE.

## Teaser

Sepsis drives brain microglia to eliminate complement tagged synapses resulting in neuronal damage and memory loss.

## INTRODUCTION

Sepsis is the most common cause of death in noncardiologic intensive care units worldwide with mortality rates up to 20 to 25% (*1*). Despite intensive efforts to establish specific therapy, effective sepsis treatment is still limited to early antibiotic treatment and support of organ function. During the course of sepsis, approximately 50% of patients develop sepsis-associated encephalopathy (SAE). The acute phase of SAE is characterized by symptoms of delirium, such as agitation, hallucinations, reduced concentration along with somnolence, and coma (*2*). Furthermore, the presence of (sub)-acute encephalopathy in patients with sepsis is strongly associated with increased mortality rates (*3*, *4*). In sepsis survivors, chronic neurocognitive impairment is one of the most frequent long-term sequelae (*5*) characterized by general memory dysfunction, attention deficits, reduced verbal fluency, and impaired executive function (*6*). Because of increasing incidence and reduced mortality in sepsis, long-term sequelae of SAE become increasingly more relevant. Despite its high clinical relevance, the pathomechanisms of SAE are still incompletely understood. A combination of pathogenic mediators has been postulated, e.g., ischemic/hemorrhagic lesions, altered microcirculation and cellular metabolism, neuroinflammation with microglia activation, blood-brain barrier dysfunction, and excitotoxicity (*2*, *4*). In particular, infiltration of peripheral immune cells into the central nervous system (CNS) and subsequent activation of CNS-resident microglia is considered to contribute to acute and chronic brain dysfunction (*7*–*9*).

Microglia are CNS-resident macrophages, and in pathological conditions, microglia undergo morphological and transcriptional transformations enabling phagocytosis, antigen presentation, and production of inflammatory mediators, e.g., cytokines and chemokines (*8*, *10*). During the physiological state, they are important mediators of tissue homeostasis, synaptic plasticity, and synaptic function (*11*, *12*). Microglia surveillance and microglia-mediated synaptic pruning are essential mechanisms for circuit refinement during brain development (*13*–*15*). In the mature CNS, synaptic pruning by microglia maintains synaptic turnover, eliminates unnecessary synapses, and establishes previously non-existing neuronal circuits (*15*). It is well known that microglia-mediated synaptic pruning is pathologically up-regulated during neurodevelopmental and neurodegenerative diseases (*14*). Recent studies on inflammatory and neurodegenerative brain disease models unveiled that synapses are tagged with the complement factor C1q leading to the initiation of the complement pathway and microglia phagocytosis of C1q- and C3-tagged synapses mediated by complement receptors located on the microglia surface (*16*–*20*). Microglia proliferation, differentiation, and survival are highly dependent on colony-stimulating factor 1 receptor (CSF1-R), opening an interventional opportunity through pharmacological inhibition of CSF1-R, which results in a pronounced microglia depletion (*21*). Pharmacological targeting of pathologically activated microglia has been shown to be effective in models for neurodegenerative diseases and therefore might be of therapeutic value (*22*, *23*).

Here, we aimed to investigate the role of microglia and pathogenic immune-neuronal interactions underlying neurocognitive dysfunction in SAE. We uncovered a key role of complement activation in SAE pathophysiology leading to aberrant synaptic tagging followed by microglia-dependent synaptic pruning. These changes are responsible for neuronal and cognitive dysfunction as pharmacological microglia depletion resulted in decreased C1q levels and improvement of neurocognitive function.

## RESULTS

### Human and murine hippocampal microglia are activated in the acute phase of SAE

**Fig. 1.**
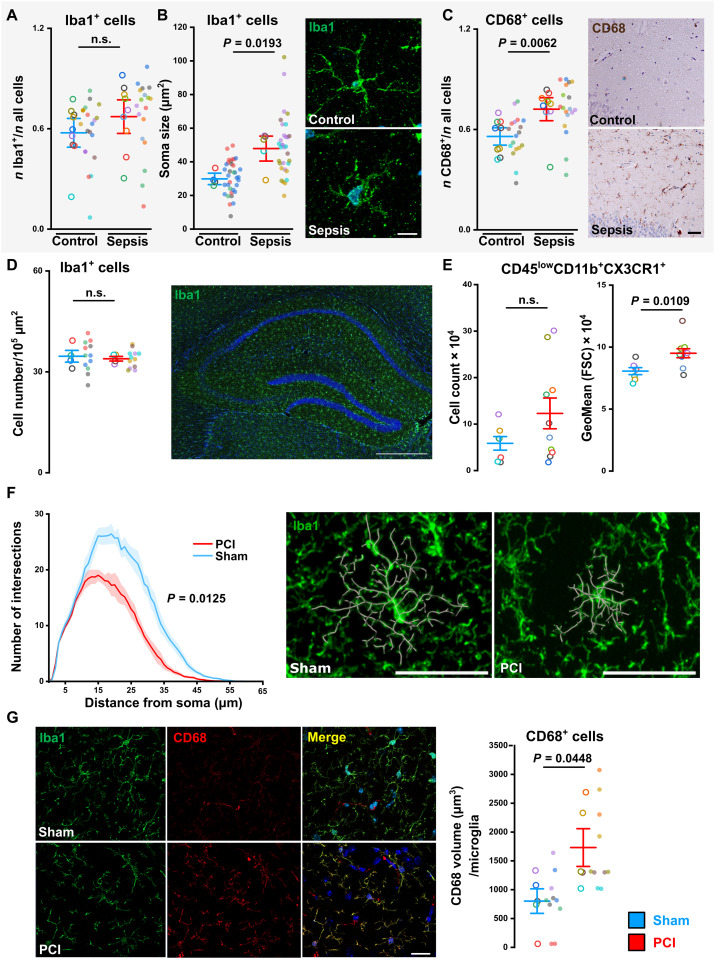
Microglia activation in the acute phase of SAE. (**A**) Ratio between Iba1-positive microglia and all cells per image in human hippocampal postmortem tissue (*n* = 9 per group; two-tailed Student’s *t* test). (**B**) Analysis of soma size of Iba1-positive microglia in human CA1 hippocampal postmortem tissue (control, *n* = 4 and sepsis: *n* = 5; 3 to 16 images per patient; two-tailed Student’s *t* test); representative image of microglia in control and patients with sepsis. Scale bar, 10 μm. (**C**) Ratio between CD68^+^ cells and all cells per image in hippocampus of patients with sepsis compared to control (*n* = 9 per group; Mann-Whitney test); representative image of CD68^+^ staining. Scale bars, 50 μm. (**D**) Cell count of Iba1-positive microglia in mice 3 days following sepsis induction (mice, *n* = 4 per group; images, *n* = 3 per mouse; two-tailed Student’s *t* test); representative confocal image of the hippocampus (sagittal section) stained with Iba1 and DAPI (4′,6-diamidino-2-phenylindole). Scale bar, 500 μm. (**E**) FACS analysis of CD45^low^CD11b^+^CX3CR1^+^ cells showing absolute cell count and geometric mean (GeoMean) of the forward scatter (FSC) representing the cell size (sham, *n* = 7; PCI, *n* = 10; two-tailed Student’s *t* test). (**F**) Sholl analysis of hippocampal Iba1^+^ cells in sham- and PCI-treated mice at day 3 after sepsis induction [*n* = 4 per group; two-way analysis of variance (ANOVA) repeated measures]; representative image with highlighted ramifications of microglia processes in sham and sepsis mice. Scale bar, 50 μm. (**G**) Immunofluorescence staining of Iba1 and CD68 in hippocampus. Scale bar, 20 μm. Quantification of CD68 volume per microglia (*n* = 5 per group; images, *n* = 2 per mouse; two-tailed Student’s *t* test). Data are presented as means ± SEM. Individual values are presented as small dots, and each circle represents an average of one mouse; individual values and averages are color coded. Gray shaded background [in (A to C)] indicates human data. n.s., not significant.

We compared microglia activation in the acute phase of SAE in human postmortem hippocampi of a patient cohort who died of sepsis with an age- and gender-matched control group with other causes of death (table S6). We found an enhanced soma size of Iba1-positive cells and an increase of the lysosomal and activation marker CD68 in the CA1 region of hippocampal tissue in the sepsis cohort, whereas the proportion of Iba1-positive cells was unchanged (Fig. 1, A to C), comparable to previous studies (*24*, *25*). To model severe polymicrobial sepsis in mice, an adjusted version of the standardized peritoneal contamination and infection (PCI) model with consecutive antibiotic rescue treatment was used, which resulted in a long-term survival rate of approximately 50% (fig. S1A and Materials and Methods). Accordingly, we observed an increased soma size of microglia in the hippocampal CA1 region 3 days after PCI, whereas we did not detect an increase of absolute microglia or macrophages/monocytes count (Fig. 1, D and E, and fig. S1, B and C), thus corroborating our findings in postmortem human samples. Moreover, Sholl analysis of hippocampal microglia revealed a less branched morphology in PCI mice, indicating an activated state (Fig. 1F). Colocalization analysis of Iba1 and CD68 showed an increase of activation marker CD68 per microglia in the hippocampus during experimental sepsis (Fig. 1G).

### Acute and persistent transcriptional changes and activity patterns of microglia following experimental sepsis

**Fig. 2.**
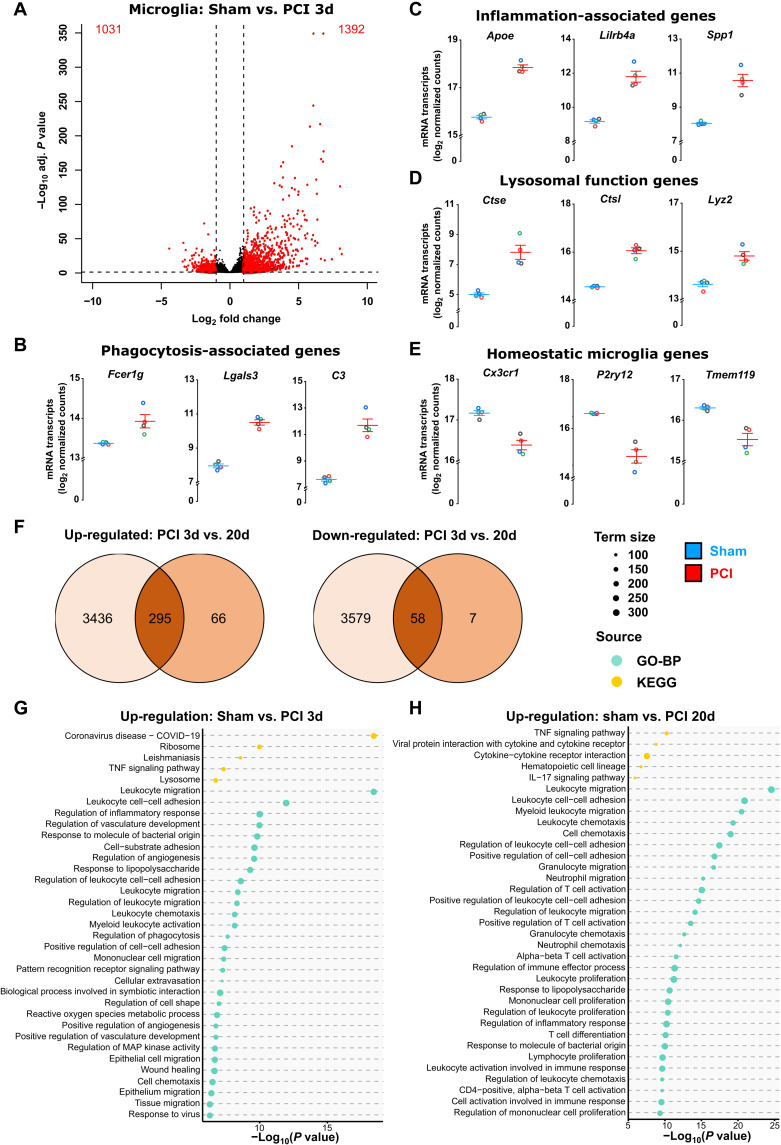
Short- and long-term microglia transcriptional changes in SAE. (**A**) Volcano plot of gene expression comparing microglia isolated of sham- and PCI-treated animals showing 1392 up-regulated (log_2_FC >1, *P*_adj_ < 0.05) and 1031 (log_2_FC < −1, *P*_adj_ < 0.05) down-regulated genes at day 3 following sepsis induction (*n* = 4 per group). (**B** to **E**) Expression of DEGs associated to phagocytosis, inflammation, lysosomal function, and homeostasis of isolated microglia after PCI- and sham-treated animals at day 3 following sepsis induction (*n* = 4 per group). (**F**) Venn diagram of DEGs in microglia comparing day 3 (*n* = 4 per group) and day 20 (*n* = 3 per group) following sepsis induction. (**G** and **H**) Enrichment analysis of top 25 up-regulated GO-BP terms and top 5 up-regulated KEGG pathways comparing PCI versus sham at day 3 (G) (*n* = 4, *P*_adj_ < 0.05) and day 20 (H) (*n* = 3, *P*_adj_ < 0.05) following sepsis induction. Data are presented as means ± SEM. Each circle represents one mouse.

To gain insight into the time course of sepsis-induced alterations of the microglial transcriptome, we next performed RNA sequencing on isolated CD11b^+^ cells 3 and 20 days after PCI. Fluorescence-activated cell sorting (FACS) analysis confirmed that isolated cells were CD45^low^CD11b^high^CX3CR1^+^ microglia (sham, 97.6% purity; PCI, 94.8% purity; fig. S2A). Three days after PCI, whole transcriptome analysis revealed a total number of 7368 differentially expressed genes (DEGs), with 1392 up-regulated and 1,031 down-regulated genes when applying log_2_FC >1 and <−1, respectively (Fig. 2A). The top 40 DEGs are shown in the heatmap (fig. S2B). We found induction of genes associated to lysosomal function (e.g., *Ctse*, *Ctsl*, and *Lyz2*), phagocytosis (e.g., *Fcer1g*, *Lgals3*, and *C3*), and inflammation (e.g., *Apoe*, *Lilrb4*, and *Spp1*), whereas homeostatic microglia genes including *Cx3cr1*, *P2ry12*, and *Tmem119* were suppressed, providing a transcriptional basis for the phenotypic change toward an activated microglia state (Fig. 2, B to E). Furthermore, among the microglia sensome (a characteristic repertoire of proteins allowing microglia to sense and respond to pathogens and to exogenous/endogenous ligands) (*26*, *27*), we found up-regulated genes encoding for proteins of phagocytosis, proteins for sensing bacterial/fungal ligands, and for complement receptors (fig. S2C).

Previous studies using systemic lipopolysaccharide (LPS) application for modeling endotoxemia described only a transient microglia activation in the acute phase without ongoing transcriptional changes at day 7 (*28*, *29*). We here performed RNA sequencing analysis in isolated microglia at even a later time point 20 days following PCI. The polymicrobial sepsis model, which more directly reflects human sepsis (*30*), resulted in persistent proinflammatory transcriptomic changes in microglia. Twenty days after PCI, 361 DEGs were still up-regulated, of which the top 40 DEGs are presented in the heatmap (Fig. 2F and fig. S2D). Comparing PCI-induced up-regulated genes of isolated microglia at days 3 and 20 demonstrated an overlap of 295 up-regulated and 58 down-regulated DEGs. Among these, we found up-regulated genes encoding for inflammation and phagocytosis associated proteins (e.g., *Apoe*, *Lgals3*, *Trem1*, and *C3*), indicating a prolonged transcriptional activation of microglia for at least 20 days following bacterial sepsis (Fig. 2F and data file S1).

In addition, at day 3 after PCI pathway enrichment analysis of all 7368 DEGs (3637 down-regulated and 3731 up-regulated) resulted in enrichment for Gene Ontology Biological Process (GO-BP) term including “leukocyte migration,” “regulation of inflammatory response,” and “response to molecule of bacterial origin” underlining activation of microglia after PCI. Kyoto Encyclopedia of Genes and Genomes (KEGG) pathway analysis revealed an up-regulation of the category “lysosome” (Fig. 2G), which is in line with increased signal of the lysosomal marker CD68 in immunohistochemistry (Fig. 1G). In contrast to LPS-induced endotoxemia (*28*), GO terms representing processes of inflammation-induced microglia activation were still up-regulated in gene enrichment analysis of isolated microglia at day 20 (Fig. 2H). In addition, GO terms “cilium organization,” “microtubule bundle formation,” and “axoneme assembly” were down-regulated (fig. S2E), thus corroborating the findings of an amoeboid microglia morphology with reduced microglial ramification (Fig. 1F). Together, we found ongoing neuroinflammation and microglia activation in the PCI model not only in the acute phase but also at later stages of SAE.

### Sepsis leads to neuronal damage and microglia-induced synaptic pruning

**Fig. 3.**
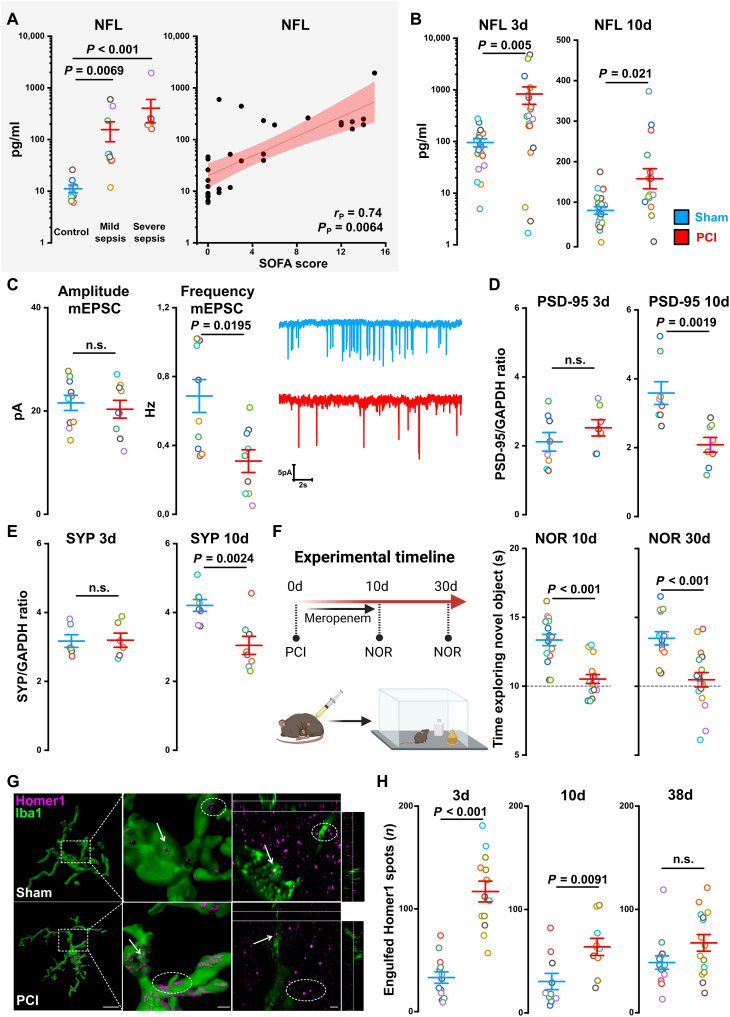
Neuronal damage and microglia-induced synaptic pruning in SAE. (**A**) Measurement of Nfl in control patients and patients with mild or severe sepsis (*n* = 10 per group; one-way ANOVA with Bonferroni’s multiple comparisons). Correlation analysis between serum Nfl and SOFA score (*n* = 30; Pearson correlation). (**B**) Measurement of Nfl in serum of PCI- and sham-treated animals at day 3 [*n* = 19 per group; Mann-Whitney *U* (MWU) test] and day 10 (sham, *n* = 21; PCI, *n* = 18; two-tailed Student’s *t* test) following sepsis induction as a marker of neuronal injury. (**C**) Patch-clamp recordings of hippocampal CA1 pyramidal cells and analyses of mEPSC in sham- and PCI-treated animals 8 weeks after PCI (*n* = 9 to 10 cells per group; MWU test). (**D** to **E**) Quantitative analysis of hippocampal pre- [synaptophysin (SYP)] and postsynaptic (PSD-95) proteins at day 3 (sham, *n* = 7; PCI, *n* = 7; two-tailed Student’s *t* test) and day 10 (*n* = 8 per group; two-tailed Student’s *t* test) comparing sham- and PCI-treated animals. (**F**) NOR test at day 10 (sham, *n* = 7; PCI, *n* = 11; two-tailed Student’s *t* test) and day 30 (sham, *n* = 7; PCI, *n* = 10; two-tailed Student’s *t* test) in sham- and PCI-treated animals. (**G**) Analysis of microglia-induced synaptic pruning. Representative three-dimensional (3D) reconstruction of microglia Airyscan imaging (left) (middle, higher magnification). Arrows indicate engulfed Homer1 spots, and circles show microglia attached Homer1 spots. Right: Individual image sections. Scale bar, 10 μm. (**H**) Quantitative analysis of microglia engulfed Homer1 spots on day 3 (*n* = 13; two-tailed Student’s *t* test), on day 10 (*n* = 10; MWU test), and on day 38 (*n* = 15; two-tailed Student’s *t* test) following PCI induction or sham injection. Data are presented as means ± SEM. Gray shaded background [in (A)] indicates human data.

To assess neuronal damage in a cohort of patients with acute sepsis and in PCI animals, we performed single molecular arrays. We measured serum concentrations of neurofilament light chains (Nfl), tau protein, and ubiquitin C-terminal hydrolase L1 (UCH-L1) as validated biomarkers of neuronal injury (*31*, *32*) in patients with mild and severe sepsis [mild, sequential organ failure assessment (SOFA) score ≤ 8; severe, SOFA score > 8; table S7]. Nfl, UCH-L1, and tau protein levels showed an increase correlated to disease severity in the sepsis patient cohort indicating neuronal and CNS injury (Fig. 3A and fig. S3, A and C). Accordingly, in PCI mice, we found an increase of Nfl at days 3 and 10 after PCI (Fig. 3B) and an increase of UCH-L1 and tau protein at day 3 (fig. S3D). To differentiate neuronal damage in mice more specifically, we next investigated hippocampal synaptic injury after PCI. Long-term synaptic loss was evidenced functionally using whole-cell patch-clamp recordings in CA1 pyramidal neurons, demonstrating reduced frequency of quantal excitatory postsynaptic currents (miniature; mEPSCs) with unchanged peak amplitudes up to 50 days after PCI (Fig. 3C). Protein analysis using capillary western immunoassay revealed a decrease of the pre- and postsynaptic markers synaptophysin and postsynaptic density protein 95 (PSD-95) at day 10 after PCI but not in the acute phase 3 days after PCI (Fig. 3, D and E, and fig. S4). Novel object recognition (NOR) test at days 10 and 30 after PCI showed a pronounced memory dysfunction after sepsis as a behavioral correlate to the hippocampal synapse loss (Fig. 3F).

On the basis of these observations, we hypothesized that PCI-activated microglia induce synaptic pruning finally leading to synaptic loss. We therefore performed colocalization analysis of the synaptic marker protein Homer1 and the microglia marker Iba1. High-resolution three-dimensional (3D) Airyscan imaging revealed enhanced engulfment of synapses (Homer1) in microglia at the acute and post-acute state on days 3 and 10 but no increase at day 38 following sepsis induction (Fig. 3, G and H). Together, these data indicate severe neuronal injury following sepsis and show synaptic pruning induced by activated microglia.

### Transcriptional profile patterns of whole hippocampus indicate synaptic damage and involvement of the complement pathway

**Fig. 4.**
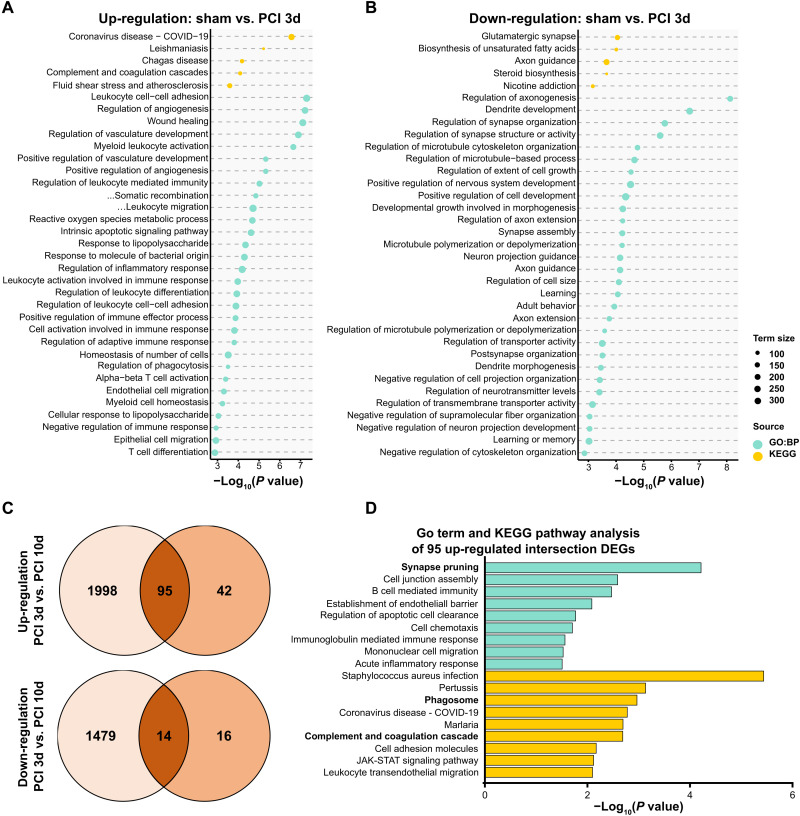
Transcriptional profile of hippocampal tissue indicating neuroinflammation and synaptic damage. (**A** and **B**) Enrichment analysis of top 25 up-regulated GO-BP terms and top 5 up-regulated KEGG pathways for PCI versus sham (*n* = 4 *P*_adj_ < 0.05) in whole hippocampus tissue at day 3 following sepsis induction. (**C**) Venn diagram of DEGs of hippocampal tissue comparing day 3 (*n* = 4 per group) and day 10 (*n* = 4 per group) following sepsis induction. (**D**) Top nine GO-BP and top nine KEGG pathway analysis of 95 up-regulated intersection genes comparing PCI day 3 (*n* = 4, *P*_adj_ < 0.05) and PCI day 10 (*n* = 3, *P*_adj_ < 0.05) following sepsis induction.

Next, we aimed at mechanistic insight into the immune-neuronal interaction ultimately resulting in synaptic loss. We therefore performed RNA sequencing of hippocampal tissue on days 3 and 10 following PCI, which was validated using quantitative polymerase chain reaction (fig. S5C). Enrichment maps and functional enrichment analysis of DEGs at day 3 after PCI revealed that sepsis resulted in up-regulation of GO-BP categories including “myeloid leukocyte activation,” “regulation of innate immune response,” and “cytokine-mediated signaling pathway,” again demonstrating a highly inflammatory state in the hippocampus (Fig. 4A and fig. S5A). Our results of neuronal/synaptic damage and impaired learning (Fig. 3, B to F) were reflected in down-regulated GO-BP categories such as “dendrite development,” “regulation of synapse structure or activity,” “learning,” and “learning and memory” (Fig. 4B and fig. S5A). KEGG pathway analysis included “complement and coagulation cascades” within the top five up-regulated pathways, indicating an involvement of the complement pathway early in sepsis (Fig. 4A). Further RNA sequencing analysis at day 10 still detected 137 up-regulated and 30 down-regulated DEGs. Further KEGG pathway analysis revealed a prolonged up-regulation of the category complement and coagulation cascades (fig. S5B). Comparing both time points, days 3 and 10, showed an intersection of 95 up-regulated genes (Fig. 4C). GO term and KEGG pathway analysis of these 95 up-regulated genes revealed categories including “synapse pruning,” “phagosome,” and “complement and coagulation,” suggesting a sustained synapse removal and involvement of the complement system during SAE (Fig. 4D and data file S2).

**Fig. 5.**
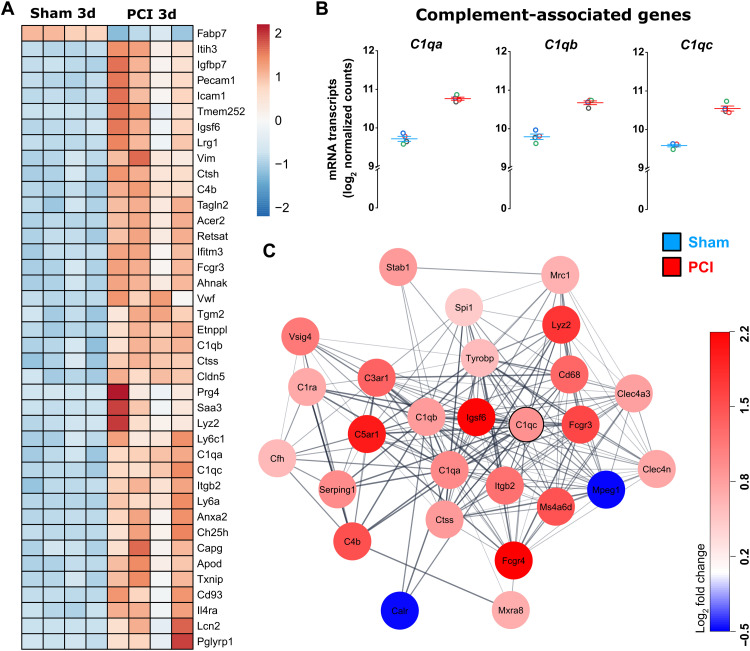
Involvement of complement factor C1q in SAE. (**A**) Expression heatmap of top 40 DEGs of hippocampal tissue in PCI- and sham-treated animals at day 3 (*n* = 4, *P*_adj_ < 0.05). The color scale represents the gene-wise *z* score calculated from normalized gene expression levels. (**B**) Expression of DEGs encoding for complement factor C1q in hippocampal tissue of PCI- and sham-treated animals at day 3 following sepsis induction. (**C**) STRING network showing protein-protein interactions of DEGs in hippocampal tissue associated with complement factor C1q in PCI- and sham-treated animals at day 3 following sepsis (*n* = 4; medium-confidence cutoff of 0.4).

On the basis of these results, we next investigated the involvement of the complement system in synaptic pathology. Previous studies in disease models with activation of the CNS innate immune system provided evidence that complement factors C1q and C3 can directly tag synapses and mediate microglia engulfment (*17*, *18*). Among the top 40 DEGs analyzed by RNA sequencing of whole hippocampus tissue at day 3 following sepsis induction, we observed up-regulation of *C1qa*, *C1qb*, and *C1qc* encoding for complement factor C1q (Fig. 5, A and B). A STRING network analysis of C1qc revealed up-regulation of interacting proteins for other complement factors (e.g., C1qa, C1qb, C4b, and C3ar1), microglia markers (e.g., spi1/PU.1), and lysosomes/phagocytosis markers (e.g., Lyz2, Ctss, Tyrobp, and CD68) (Fig. 5C), again strengthening the hypothesis of complement- and microglia-dependent synaptic damage.

### Sepsis leads to synapse tagging by complement factor C1q and its phagocytosis by microglia

**Fig. 6.**
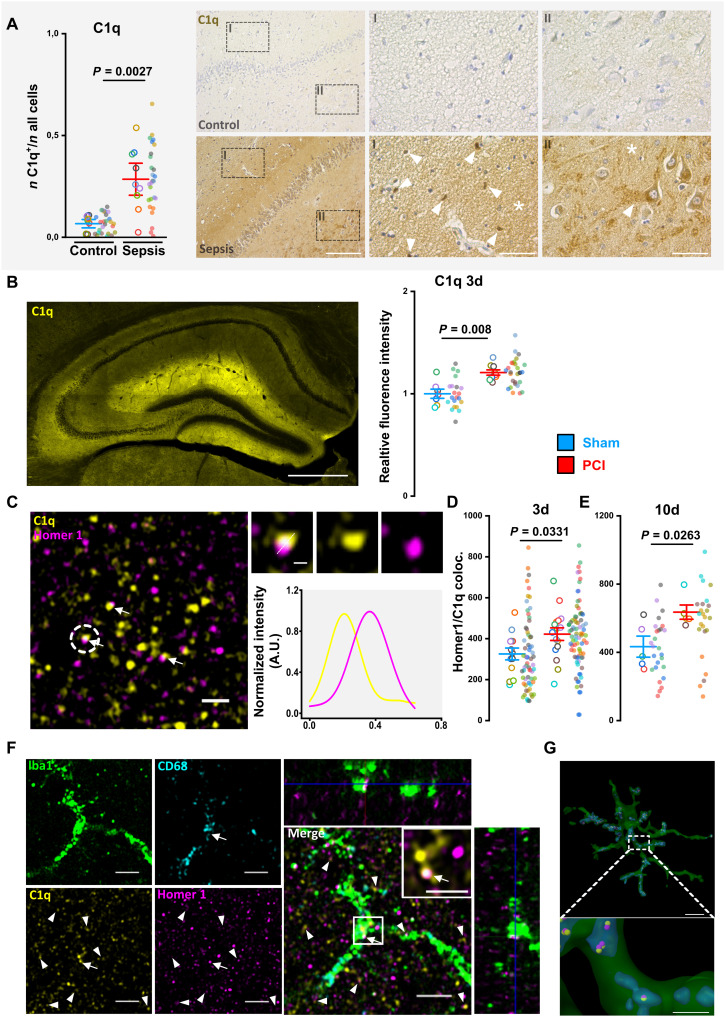
Sepsis leads to synapse tagging by complement factor C1q and its opsonization by microglia. (**A**) C1q staining in human postmortem hippocampus (I, CA1; II, gyrus dentatus) comparing sepsis and control patients (*n* = 9 per group; Mann-Whitney test). Representative images showing cell associated (arrow) and neuronal C1q staining patterns and a diffuse staining of the parenchyma (asterisk). Scale bars, 200 μm (left), 50 μm (insets). (**B**) C1q expression in the hippocampus of PCI- and sham-treated animals at day 3 following sepsis. Representative confocal image of the hippocampus (PCI animal; midsagittal section) and analysis of C1q immunofluorescence (sham, *n* = 7; PCI, *n* = 9; images, *n* = 3 per mouse; two-tailed Student’s *t* test). Scale bar, 500 μm. (**C**) Colocalization analysis of C1q and Homer1 at (**D**) day 3 (3d; sham, *n* = 13; PCI, *n* = 16; images, *n* = 5 per mouse; two-tailed Student’s *t* test) and (**E**) day 10 (10d; *n* = 5 per group; images, *n* = 5 per mouse; two-tailed Student’s *t* test) in the hippocampus of sham- and PCI-treated animals. Arrows indicated C1q-tagged synapses. Scale bars, 1 μm and 200 nm (inset). (**F**) Representative high-resolution Airyscan image of Iba1, CD68, C1q, and Homer1 showing a colocalization of C1q-tagged synapses in the lysosomes of a microglia after PCI; the inset shows only C1q/Homer 1 colocalization. Scale bars, 2 μm and 1 μm (inset). (**G**) 3D reconstruction of microglia shown in (F). green, microglia; blue, lysosome; yellow, C1q; magenta, Homer1. Note the localization of C1q-tagged Homer1 spots within microglial lysosomes. Scale bar, 8 μm. Data are presented as means ± SEM. Gray shaded background [in (A)] indicates human data. Individual values are presented as small dots, and each circle represents an average of one mouse; individual values and averages are color coded. A.U., arbitrary units.

To validate the involvement of C1q in human sepsis, we performed immunohistological staining in postmortem hippocampus samples of patients with sepsis. Here, we found an increased cell-associated C1q staining pattern (Fig. 6A). To test the involvement of C1q in PCI-induced synaptic pathology in mice, we performed immunofluorescence stains in mouse brain tissue. We found increased hippocampal C1q signal on day 3 after PCI (Fig. 6B). High-resolution colocalization analysis of C1q and Homer1 in the CA1 region of the hippocampus revealed a marked increase of C1q-tagged synapses in mice following PCI on days 3 and 10 (Fig. 6, C to E). To further detail the subcellular localization of C1q labeled synapses in microglia, we performed a costaining of Iba1, CD68, Homer1, and C1q. Here, we found C1q-labeled Homer1-positive synapses colocalizing with the lysosomal marker CD68 in Iba1-positive microglia of the CA1 region (Fig. 6, F and G). Together, these findings suggest that C1q tagging of synapses leads to the engulfment of synapses by activated microglia during the course of SAE, which is then followed by lysosomal degradation.

### Microglia depletion reduces C1q in the hippocampus and improves neurocognitive outcome after sepsis

**Fig. 7.**
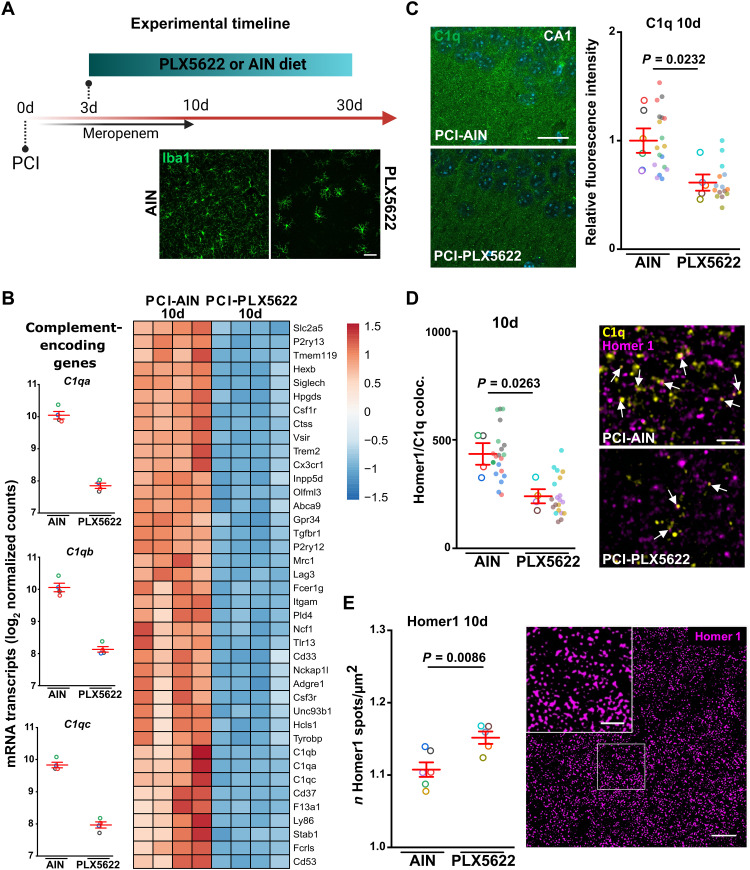
Microglia depletion decreases C1q expression and increases synapse number after sepsis. (**A**) Schematic timeline of the experiments. Nine to 13-week-old male mice were randomized to PCI or sham treatment. Both groups received meropenem treatment for 10 days. At day 3 following sepsis induction or sham treatment, AIN control diet or PLX5622 diet was started until the end of experiment. Insets show Iba1-positive microglia in the CA1 region of mice 10 days after PCI and AIN (left) or PLX5622 (right) treatment, respectively. Scale bar, 50 μm. (**B**) Expression of DEGs encoding complement factor C1q in PCI-AIN– and PCI-PLX5622–treated animals at day 10 following sepsis induction (*n* = 4 per group). Expression heatmap of top 40 DEGs of hippocampal tissue of PCI-AIN–treated versus PCI-PLX5622–treated animals (*n* = 4, *P*_adj_ < 0.05). The color scale represents the gene-wise *z* score calculated from normalized gene expression levels. (**C**) Representative image (scale bar, 20 μm) and quantification of C1q immunoreactivity in PCI-AIN and PCI-PLX5622–treated animals at day 10 in the CA1 region of the hippocampus (PCI-AIN, *n* = 6; PCI-PLX5622, *n* = 5; analyzed images, *n* = 3 per mouse; two-tailed Student’s *t* test). (**D**) Colocalization analysis of C1q and Homer1 at day 10 (10d) after sepsis (PCI-AIN, *n* = 4; PCI-PLX5622, *n* = 4; images, *n* = 5 per mouse; two-tailed Student’s *t* test) in the CA1 region of the hippocampus. Arrows indicate C1q-tagged synapses. Scale bar, 2 μm. (**E**) Superresolved lattice-SIM (structured illumination microscopy) images of Homer1 spots at day 10 (10d) after sepsis (PCI-AIN, *n* = 6; PCI-PLX5622, *n* = 5; two-tailed Student’s *t* test) in the CA1 region of the hippocampus. Scale bars, 5 and 2 μm (inset). Data are presented as means ± SEM. Individual values are presented as small dots, and each circle represents an average of one mouse; individual values and averages are color coded.

**Fig. 8.**
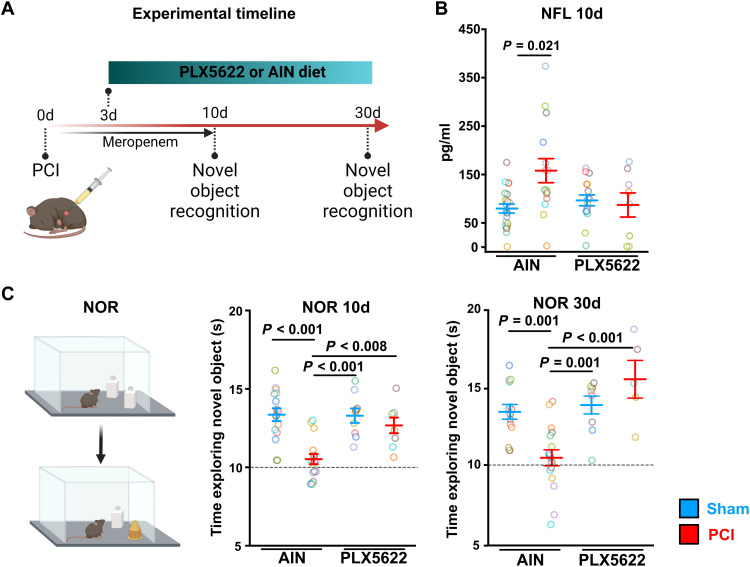
Microglia depletion improves neurocognitive outcome after sepsis. (**A**) Schematic timeline of the experiments. Nine- to 13-week-old male mice were randomized to PCI or sham treatment. Both groups received meropenem treatment for 10 days. At day 3 following sepsis induction or sham treatment, AIN control diet or PLX5622 diet was started until the end of the experiment. NOR tests were performed at the indicated time points. (**B**) Measurement of serum Nfl as a marker of neuronal injury in serum of PLX5622- or AIN-treated animals at day 10 after sepsis (sham-AIN, *n* = 21; PCI-AIN, *n* = 18; sham-PLX5622, *n* = 16; PCI-PLX5622, *n* = 8; one-way ANOVA with Bonferroni’s multiple comparisons test). (**C**) NOR at day 10 (sham, *n* = 14; PCI, *n* = 22; sham-PLX5622, *n* = 9; PCI-PLX5622, *n* = 8) and day 30 (sham, *n* = 14; PCI, *n* = 17; sham-PLX5622, *n* = 9; PCI-PLX5622, *n* = 5; Kruskal-Wallis ANOVA with Dunn’s method for multiple comparisons test) following sepsis induction in PLX5622- and AIN-treated animals. Data are presented as means ± SEM. Individual values are presented as small dots, and each circle represents an average of one mouse; individual values and averages are color coded.

Microglia are known to be an essential source of C1q in CNS pathology (*33*, *34*) and mediate synaptic pruning after complement tagging (*17*, *18*). We therefore depleted microglia by supplying diet that contained the CSF1-R inhibitor PLX5622 (1200 mg/kg) or control diet (AIN; Figs. 7A and 8A). Treating mice with PLX5622 (1200 mg/kg) in a preventive manner 7 days before PCI induction resulted in the death of all PCI animals (fig. S6A). Therefore, we performed PLX5622 application in a therapeutic regime starting at day 3 after PCI, which did not have an impact on the overall survival after sepsis (fig. S6B). PLX5622 treatment for 7 days greatly reduced the number of microglia in the CA1 region in PCI and control mice (fig. S6C). Accordingly, after 7 days of PLX5622 treatment, FACS analysis revealed a profound reduction of CD45^low^CD11b^high^CX3CR1^+^ marked resident hippocampal microglia in sham- and PCI-treated animals (fig. S6D). The number of CD45^high^CD11b^+^CX3CR1^+^F4/80^+^ macrophages and CD45^high^CD11b^high^CX3CR1^+^F4/80^−^ monocytes were increased following sepsis induction but were not affected by pharmacological CSF1-R inhibition (fig. S6, E and F). Comparing hippocampal tissue RNA sequencing results of PLX5622- and AIN-treated mice 10 days after PCI (i.e., after 7 days of PLX5622 treatment), we found down-regulation of *C1qa*, *C1qb*, and *C1qc* (Fig. 7B) and of genes encoding for phagocytosis proteins (e.g., *Trem2*, *Tyrobp*, and *Fcer1g*; fig. S6G), as well as down-regulation of neuroinflammatory GO term categories (fig. S7). Microglia depletion also resulted in reduced C1q signal in immunohistochemical analysis of the hippocampus 10 days after sepsis induction (Fig. 7C). Similar effects on C1q transcriptional expression and C1q signal could be found in microglia-depleted non-PCI animals (fig. S8, A to C), thus supporting previous findings of microglia being a source of C1q (*33*). Further high-resolution colocalization analysis of C1q and Homer1 in the CA1 region of the hippocampus revealed a decrease of C1q-tagged synapses after PLX5622 treatment (Fig. 7D). We could not detect any differences in microglia synapse engulfment (Homer1) in the few remaining microglia at day 10 after sepsis (fig. S9A). However, we found a substantially increased synapse density by measuring Homer1 signal in PCI mice after PLX5622 treatment at day 10 (Fig. 7E). Moreover, we detected an increased mature mushroom spine density in biocytin-filled neurons of the hippocampal CA1 region in microglia-depleted mice in late stages after sepsis induction (fig. S9, B and C). Ten days after PCI, Nfl levels were still increased in PCI mice fed with control diet. Treatment with PLX5622 was able to prevent Nfl level increase, indicating a neuroprotective effect (Fig. 8B). Last, using the NOR and Barnes maze test, we investigated the therapeutic potential of microglia depletion on neurocognitive deficits of PCI mice. Notably, PLX5622 treatment starting at day 3 after PCI improved learning deficits compared to PCI animals with control diet in NOR and Barnes maze test, whereas exploration and locomotor behavior was unaffected (Fig. 8C and fig. S9, D and E), strengthening the link between sepsis-induced cognitive dysfunction and inflammation-associated microglia activation and microglia-induced synaptic pruning.

### Intrahippocampal injection of a C1q-blocking antibody reduces synaptic pruning after sepsis

**Fig. 9.**
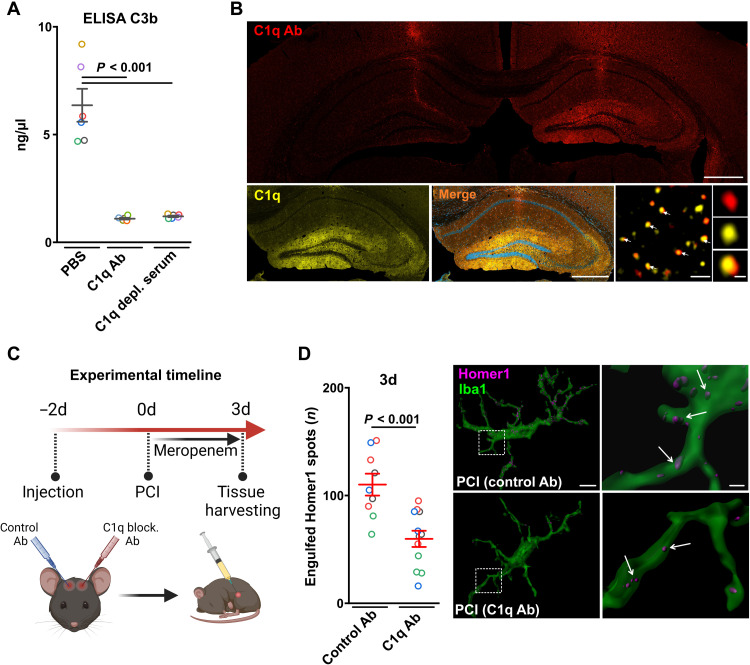
C1q-blocking antibody decreases C1q-induced synapse pruning after sepsis. (**A**) Validation of the blocking functionality of the C1q-blocking antibody (C1q Ab) by ELISA testing C1q-dependent classical complement activation using complement active human serum. Downstream C3b concentration is determined in three different groups: phosphate-buffered saline (PBS), positive control group; C1q Ab, blocking antibody; C1q-depleted serum, negative control (*n* = 6 per group, one-way ANOVA with Bonferroni’s multiple comparisons test). (**B**) Top: Representative confocal image of a coronal brain section showing both hippocampi after in vivo microinjection (left: control antibody; right: C1q-blocking antibody) demonstrating a locally restricted distribution of C1q antibody in the respective hippocampus at the site of C1q expression. Scale bar, 500 μm. Bottom images left and middle: Colocalization of commercial C1q detection antibody (Abcam) and in vivo injected C1q-blocking antibody showing specificity of the blocking antibody. Scale bar, 500 μm. High-resolution imaging (right) reveals colabeling of single C1q-positive spots [scale bars, 5 μm (left) and 1 μm (right)]. (**C**) Schematic timeline of the experiments. Nine- to 13-week-old male mice received intrahippocampal injection of blocking C1q antibodies into one hemisphere and control antibodies into the other hemisphere to avoid any potential interindividual differences. Two days after, experimental sepsis was induced by PCI followed by meropenem treatment until the end of experiment. At day 3 following PCI, brain tissue was harvested for analysis. (**D**) Analysis of microglia-induced synaptic pruning after intrahippocampal C1q blocking (*n* = 12) or control antibody injection (*n* = 9). Representative 3D reconstruction of microglia Airyscan imaging [scale bar, 5 μm (left) and 1 μm (right, higher magnification)]. Arrows indicate engulfed Homer1 spots. Data are presented as means ± SEM. Individual values are color coded.

To test a direct link between C1q and synaptic pruning during the course of sepsis, we performed a set of in vitro and in vivo experiments using a specific C1q-blocking antibody to inhibit C1q and its downstream mechanisms of synaptic C3b opsonization. To test the blocking functionality of the C1q antibody in vitro, we performed enzyme-linked immunosorbent assay (ELISA) measuring C3b concentration following classical complement activation in normal human serum (NHS) by coated immunoglobulin M (IgM). Preincubation with C1q-blocking antibody or the use of C1q-depleted serum prevented C3b production, indicating a high functionality of the C1q-blocking antibody (Fig. 9A). After in vivo intrahippocampal injection, the C1q-blocking antibody accumulated locally in the hippocampal regions with pronounced C1q expression and specifically bound to C1q in vivo as demonstrated by costaining with a commercial anti-C1q antibody 5 days following injection (Fig. 9B). To test the effect of the blocking antibody in SAE, we next performed intrahippocampal injection of the C1q-blocking antibody into the left hippocampus and control antibody into the right hippocampus 2 days before sepsis induction (Fig. 9C). We performed high-resolution 3D Airyscan imaging in hippocampal CA1 region at day 3 following PCI. Colocalization analysis of Iba1 and Homer1 revealed a decreased engulfment of Homer1 synapses in microglia of PCI animals after injection of the C1q-blocking antibody as compared to the control antibody (Fig. 9D). Together, our data show that specifically targeting C1q results in decreased number of phagocytosed synapses by activated microglia during the course of SAE, thus demonstrating a direct link of C1q with increased synaptic pruning.

## DISCUSSION

Recent reports suggested microglia activation as a relevant pathomechanism in SAE and proposed several targets for potential intervention, e.g., increased cytokine expression (*35*), NLR Family Pyrin Domain Containing 3 (NLRP3) inflammasome formation (*36*), or complement system activation (*37*). Here, we uncovered an early involvement of the complement system as a key player in the pathomechanism of hippocampal synaptic damage during polymicrobial sepsis. Our findings demonstrate that microglia are responsible for removal of C1q-tagged synapses during the course of SAE and that injection of a C1q-blocking antibody rescues microglial synapse engulfment and synapse loss. Microglia depletion in the post-acute phase of sepsis is sufficient to improve sepsis-induced cognitive decline.

We found sepsis-induced diffuse parenchymal and cell-specific (e.g., neurons and microglia) C1q deposition in postmortem patient brain sections and in PCI mouse tissue. Our data are in line with previous reports showing similar staining patterns in multiple sclerosis and Alzheimer disease patients (*38*, *39*). We provide evidence that C1q is responsible for synapse tagging after experimental sepsis and subsequent microglial phagocytosis during SAE, as synaptic pruning could be prevented by C1q neutralization with a specific blocking antibody. Similar therapeutic strategies have been used in several experimental disease models, e.g., for neurodegenerative diseases (*18*, *34*, *40*). Recently published evidence supports the hypothesis that C1q tagging is dependent on apoptotic factors in the synapse rather than on the overall complement protein level (*19*). This finding might also be applicable after sepsis, as increased neuroinflammation is likely to induce apoptotic signaling by activation of caspase 3 (*41*). Moreover, phosphatidylserine has been identified as a key mediator and an “eat me” signal on synapses. Membrane exposure of phosphatidylserine leads to C1q tagging and subsequent phagocytic removal by microglia (*20*, *42*), as we also observed during SAE.

It has been speculated that microglia and activation of the complement system mediate synaptic pruning after LPS-induced endotoxemia (*37*, *42*, *43*). Although widely accepted as a model of acute systemic inflammation (*25*), the LPS model only insufficiently describes sepsis because no replicable pathogens are applied, which maintain infection in the disease course. Thus, LPS application results in a systemic inflammatory response syndrome, rather than sepsis, with a short-lasting cytokine burst and immune-activation including very transient transcriptional changes in microglia (*26*, *28*, *29*). As we report here, the polymicrobial PCI sepsis model caused a prolonged and strong activation of microglia beyond the early stages after PCI induction. PCI led to active neuroinflammation even at day 20 after PCI including transcriptional changes indicating increased phagocytosis and inflammation-induced microglia and complement activation. In line with these observations, human autopsy results shown here and by others corroborate extensive activation of CD68-positive, phagocytotic active microglia in patients with sepsis also in advanced disease states (*24*, *44*).

Intervention in the disease course by microglia depletion resulted in a reduction of genes encoding for microglia phagocytosis proteins, e.g., *Dap12*, *CD33*, *Trem2*, and *Fcer1g*, as expected. Moreover, C1q coding genes, C1q deposition, and C1q/Homer1 colocalization in the hippocampus were decreased. We did not observe decreased engulfment of synapses in the remaining microglia in PLX5622-treated mice. However, because the overall number of microglia is markedly decreased in PLX5622-treated animals, the total number of engulfed synapses is severely reduced, leading to a higher number of Homer1 synapses and mature mushroom spines. Furthermore, directly targeting C1q with a specific C1q-blocking antibody resulted in a marked protection of synapse engulfment following experimental sepsis. Microglia are not only effectors, e.g., for synapse phagocytosis, but also an essential source for complement factors, which supports recent reports using single-nucleus RNA sequencing or conditional knockout models to demonstrate microglia-dependent complement deposition in the brain (*33*, *34*). Thus, aside from the main effect of preventing phagocytosis, reduction of microglia-dependent complement deposition might also contribute to the beneficial effect of pharmacological microglia depletion in a therapeutic strategy after sepsis onset. These target-directed therapeutic approaches are in line with preclinical studies demonstrating a beneficial effect of CSF1-R inhibitor-induced microglia depletion, e.g., in Alzheimer disease models and after traumatic brain injury (*22*, *23*). With an oral bioavailability of >30% and a depletion of microglia up to 95% in the CNS, PLX5622 might be a promising candidate with translational potential in the prevention of long-term effects of SAE (*22*).

There are some limitations in our study. CSF1-R is not only exclusively expressed on microglia but also present on other peripheral and central myeloid cells, such as monocytes and macrophages. Accordingly, we found that treating mice with PLX5622 (1200 mg/kg) in a preventive manner 7 days before PCI induction instead of starting at day 3 after PCI resulted in the death of all PCI animals. This is most likely caused by alterations and depletion of peripheral immune cells (*45*), thus leading to an incompetent host defense to bacterial pathogens in the acute phase of sepsis. The high mortality rate following pretreatment with PLX5622 limits the possibility to study the importance of microglia depletion in the very acute phase of sepsis. However, therapeutic application of PLX5622 from day 3 after sepsis induction, while still in the active phase of systemic inflammation, did not result in increased mortality and detrimental effects, thus proving its therapeutic potential in a setting closely reflecting the clinical situation of patients with sepsis. Considering our results showing a temporary increase of C1q-mediated synaptic pruning during the acute (day 3) and post-acute phase (day 10) of sepsis but not in the chronic phase (day 38), future studies should evaluate shorter and intermittent application of PLX5622 to investigate the effects of transient microglia depletion and also repopulation to test the therapeutic efficacy of a temporary limited microglia depletion in the acute and post-acute phase. Recent studies also highlighted that microglia depend more on CSF1-R signaling than peripheral immune cells and are therefore more sensitive to CSF1-R targeting (*46*). We can confirm these findings by our FACS analyses indicating that PLX5622 treatment affects other monocytic brain immune cells to a lesser degree than microglia during the course of sepsis. Alternative models, such as diphtheria toxin–induced microglia depletion can also efficiently reduce microglia expression in the brain (*47*). However, these models are associated with a potential harmful extensive cytokine release in the brain due to simultaneous ablation of all microglia at the same time point, which might interfere with the beneficial effects and the course of SAE (*47*, *48*). Furthermore, the ongoing neuroinflammation also at later time points after sepsis, might necessitate prolonged reduction of microglia, thus arguing for a long-term pharmacological intervention. As a further limiting aspect, we cannot rule out that also other mechanisms besides complement activation and microglia-induced synapse pruning, e.g., microglia-dependent cytokine signaling or NLRP3 inflammasome activation, contribute to the pathophysiology observed in SAE.

As an alternative approach, directly targeting C1q might also be a conceivable interventional strategy, as we could show here that a blocking C1q antibody can interfere with the key pathogenic event of synapse pruning. Therapeutic C1q-blocking antibodies are already tested in clinical trials for Guillain-Barré syndrome and amyotrophic lateral sclerosis (e.g., ANX005 in NCT04514367 and NCT04569435). However, in human sepsis and in our experimental sepsis model, systemic C1q inhibition would severely affect host defense and exacerbate systemic inflammation during sepsis (*49*). Therefore, it is mandatory that complement inhibition is restricted to the CNS compartment, and established C1q-blocking antibody application protocols (*34*, *40*, *50*) cannot be used in the PCI model to test a direct therapeutic effect on cognitive behavior. Direct intrahippocampal microinjections of the C1q-blocking antibody as used here are well suited to study cellular and molecular consequences of C1q- and microglia-mediated pathology at the site of injection but have clear limitations to study effects on behavior. CNS-targeted delivery of sufficient and well distributed therapeutic IgG or development of bispecific antibody constructs, e.g., “brain-shuttle” constructs (*51*) for active transport across the blood-brain barrier are important next steps for the implementation of effective and target-specific therapy in the brain.

In conclusion, our study identifies a pathogenic relevant and sustained microglia-neuron interaction during SAE. These changes induce the elimination of C1q-tagged synapses and eventually result in cognitive dysfunction. Microglia manipulation or directly targeting C1q might be a promising treatment strategy for preventing long-term neurocognitive deficits in sepsis survivors.

## MATERIALS AND METHODS

### Study design

We designed a series of studies to investigate the molecular mechanism of synapse injury in SAE and to test the hypothesis that microglia cause synapse loss by removal of C1q-tagged synapses in the hippocampus. Therefore, we first performed an observational study of human autopsy material to investigate microglia activation and C1q deposition in hippocampal tissue. Unbiased RNA sequencing analyses of hippocampal tissue and of isolated microglia derived from a murine model of polymicrobial peritoneal sepsis (PCI model) were performed to investigate transcriptional changes in SAE, thus uncovering an involvement of the complement system. Next, we designed a prospective cohort study in the PCI model to determine whether hippocampal pathology and cognitive dysfunction is associated to C1q activation and mediated by microglia-induced synaptic pruning. Last, we performed a prospective study using PLX5622 and a specific C1q-blocking antibody to test the therapeutic potential of microglia depletion, C1q inhibition, and interference with microglia-dependent synaptic pruning on hippocampal pathology and cognitive dysfunction in SAE.

Animal studies were conducted in accordance with the German animal welfare legislation and approved by the Thüringer Landesverwaltungsamt. Studies involving human subjects were approved by the Ethics Committee of the Jena University Hospital.

### Human material

Postmortem brain tissue of nine patients who died of sepsis and nine patients with a different cause of death were obtained from the Institute of Pathology at the University Hospital Jena (table S6). Patients in the sepsis group had the clinical diagnosis of sepsis, which was confirmed in the autopsy. Patients of the control group had neither clinical signs of sepsis nor any sign in the autopsy. Patient groups were gender- and age-matched, and patients with underlying neurological diseases were excluded.

Furthermore, plasma obtained from 10 control patients, 10 patients with mild sepsis (SOFA score < 8), and 10 patients with severe sepsis (SOFA score > 8) at day 3 (±1) after sepsis diagnosis were analyzed for neuronal injury markers (table S7). Patients were age-matched and consist of a subgroup of an ongoing single-center prospective cohort study (DRKS00013347, NCT03620409) (*52*). Use of all patient material was approved by the local ethics committee of the Friedrich Schiller University Jena (5276-09/17-ICROS and 2021-2423-Material).

### Animals

All experiments were performed in accordance with the ARRIVE guidelines (*53*) and the German legislation on protection of animals and with approval of the local animal welfare committee (Thüringer Landesamt für Lebensmittelsicherheit und Verbraucherschutz, 02-085/14 and UKJ-18-026). For each experiment, C57BL/6J male mice from the in-house breeding facility were used and randomly selected at the age of 10 to 13 weeks. Animals were housed under controlled/standardized day-night conditions (12 hours/12 hours) at room temperature (23° ± 1°C at 30 to 60% environmental humidity) and received a standard diet (AIN) and water ad libitum.

### PCI sepsis model and PLX5622 treatment

Sepsis was induced by the standardized and established PCI approach (*30*). Briefly, intraperitoneal injection of fecal slurry (diluted 1:4 in saline solution; 3.5 μl/g body weight) or saline solution (sham) was performed into the right lower quadrant of the abdomen with a 21-gauge cannula. To increase survival rate and ensure comparability sham- and PCI-treated animals received the same antibiotic treatment regime. Meropenem (20 mg/kg body weight) was injected subcutaneously in sham- and PCI-treated animals every 12 hours for 7 days starting at a clinical severity score (CSS) of 3 in PCI mice. On days 8 to 10, meropenem was applied every day. Thereafter, to avoid reoccurring systemic infection at later stages, enrofloxacin (Baytril 2.5%; Bayer AG, Germany) was added to the drinking water of sham- and PCI-treated animals with saccharose added in a final concentration of 2 mg/ml until the end of the experiment (*54*).

Disease course was continuously assessed using the CSS as previously described (*30*). Only mice with a cumulative 3-day CSS of over 9.5 and a cumulative 5-day CSS of over 10.5 were included in the study for further analysis to ensure adequate disease severity.

In microglia depletion experiments using PLX5622 (1200 mg/kg; Plexxikon Inc., Berkeley, CA), 3 days after PCI, the control diet AIN was exchanged to PLX5622-containing chow in the intervention group. Detailed information for the time course of PLX5622 or AIN treatment is provided in Figs. 7A and 8A. In a microglia depletion experiment with PLX5622 treatment before PCI, mice received PLX5622 treatment 7 days before PCI. Mice were weighed to assess adequate uptake of PLX5622 and AIN. At the end of the experiments, animals were sacrificed by overdosage of isoflurane (2%). Subsequently, blood was collected by right ventricular heart puncture. Brain tissue was harvested for further ex vivo experiments at the respective time points (see below).

### Generation of the blocking monoclonal anti-C1q mouse antibody with silenced Fc

For the generation of the anti-C1q mouse antibody, nucleotide sequences for the heavy (VH) and light (VL) chain of the humanized anti-C1q antibody M1 (patent WO2016073685A1) were ordered from GeneArt (Thermo Fisher Scientific, Germany). The amino acid sequences of both antibody chains are shown in Table 1. Subsequently, the V-genes were cloned into the mouse IgG2a vectors. These mouse vectors are based on the human IgG1 vectors previously published (*55*). The VH was subcloned into the pCSEHm2a.2-smFc-Xp (murine heavy chain) vector with a silenced Fc part containing the three mutations L234A, L235A, and N297A in the CH3 domain. These mutations lead to reduced mouse Fc gamma receptor complement binding (*56*). The VL was subcloned in the pCSL3mk.2-Xp (murine light chain kappa) vector. Both cloning steps were adapted for Golden Gate Assembly with Esp 3I restriction enzyme recognition sites (New England Biolabs, Germany). The assembly steps and anti-C1q mouse antibody production and purification were performed as previously described (*57*, *58*). Human embryonic kidney Expi293F cells (Thermo Fisher Scientific, Germany, #A14527, RRID:CVCL_D615) were cultured at 37°C and 110 rpm with 5% CO_2_ in Gibco FreeStyle F17 expression medium (Thermo Fisher Scientific, Germany). The medium was supplemented with 8 mM glutamine and 0.1% Pluronic F-68 (PAN Biotech, Germany). For the transfection, DNA (1:1 ratio of the vectors for IgG production) and 40-kDa PEI (Polysciences, Germany) were then mixed and incubated 25 min at room temperature before the mixture was added to the cells. After 48 hours, the culture volume was doubled by feeding HyClone SFM4Transfx-293 medium (GE Healthcare, Germany) supplemented with 8 mM glutamine. In addition, HyClone Boost 6 supplement (GE Healthcare, Germany) was added with 10% of the end volume. A week after transfection, supernatant was harvested by 15-min centrifugation at 1500*g*, and the anti-C1q antibody was purified using the Profinia System (Bio-Rad Laboratories, Germany).

**Table 1.** Amino acid sequences of M1 antibody heavy and light chain variable domains.

VH	QVQLQQPGAELVKPGASVKLSCKSSGYHFTSYWMHWVKQRPGQGLEWIGVIHPNSGSINYNEKFESKATLTVDKSSSTAYMQLSSLTSEDSAVYYCAGERDSTEVLPMDYWGQGTSVTVSS
VL_kappa_	DVQITQSPSYLAASPGETITINCRASKSINKYLAWYQEKPGKTNKLLIYSGSTLQSGIPSRFSGSGSGTDFTLTISSLEPEDFAMYYCQQHNEYPLTFGAGTKLELK

### Transcriptome expression analysis

The RNA sequencing reads were aligned to the mouse reference genome (GRCm38 with the Ensembl genome annotation release 99) using STAR 2.7.2b (parameters: --alignIntronMax 100000, --outSJfilterReads Unique, --outSAMmultNmax 1, --outFilterMismatchNoverLmax 0.04) (*59*). For each gene, all reads that map uniquely to one genomic position were counted with FeatureCounts 1.6.5 (multimapping or multi-overlapping reads were discarded; stranded mode was set to “–s 2”) (*60*). DEGs were determined with R 3.6 using the package DESeq2 1.26.0 (*61*). Only genes that have at least one read count in any of the analyzed samples of a particular comparison were subjected to DESeq2. For microglia, the sample group “sham” was compared to the group “PCI” at days 3 and 20 following sepsis induction. For hippocampus, the sample group sham was compared to the sample group PCI at days 3 and 10 following sepsis induction; the sample group PCI was compared to the sample group “PCI-PLX,” and the group sham was compared to the group “sham-PLX” in a pairwise fashion (in total, six comparisons). For each gene in each comparison, the *P* value was calculated using the Wald significance test. Resulting *P* values were adjusted for multiple testing using the Benjamini and Hochberg correction. The log_2_ fold change (log_2_FC) values were shrunk with the DESeq2 function lfcShrink(type = “normal”) to control for variance of log_2_FC estimates for genes with low read counts. Genes with an adjusted *P* value (*P*_adj_) < 0.05 were considered as DEGs. Heatmaps of selected DEGs were generated with the R package pheatmap 1.0.12. The DESeq2-normalized counts were scaled and centered for each gene (i.e., *z* score), and rows were clustered with kmeans based on the Pearson correlation distance.

GO term enrichment analysis was performed using R with R Studio. DEGs were selected with an adjusted *P* value ≤0.05 and subjected to GO term enrichment analysis using g:Profiler tool g:GOSt of the gprofiler2 package (*62*). The significance threshold was set to *P* ≤ 0.05 with the multiple correction method g:SCS. As sources, GO biological processes and the KEGG databases were chosen, while electronic GO annotation was excluded. Last, term size was limited to ≤350, and gem files were exported for downstream analysis in Cytoscape. Top GO terms were selected on the basis of the *P* value. Networks were calculated in Cytoscape with the aid of EnrichmentMap and AutoAnnotate apps. The node *q* cutoff value was set to 0.05. Venn diagrams showing the overlap of DEGs in different treatment groups were calculated and plotted with the VennDiagram package. Detailed additional information regarding the analysis pipeline is shown in Supplementary Materials and Method.

Networks of murine C1qc first neighbor interactors were constructed using the STRING protein database. The maximum additional interactors were limited to 50 with the confidence cutoff set to 0.4. The network was then reduced to proteins that are related to DEGs (*P*_adj_ ≤ 0.05), and the corresponding log_2_ fold changes were mapped to the node fill color.

### Statistics

Statistical analysis was performed with OriginPro (v. 2020b) and SigmaPlot (v. 14.5). If data were normally distributed (Shapiro-Wilk test), then two-tailed unpaired Student’s *t* test (two groups) or one-way analysis of variance (ANOVA) (more than two groups) with Bonferroni’s multiple comparisons test was used. If data were not normally distributed, Mann-Whitney test (two groups) or Kruskal-Wallis ANOVA (more than two groups) with Dunn’s method for multiple comparisons test was used. Sholl analysis and Barnes maze were tested using two-way ANOVA repeated measurements with Bonferroni’s multiple comparisons test. Outliers were identified using Grubbs’ test. If applicable, different statistical analyses are described in the respective figure legend. All graphs are represented as super-plots showing means ± SEM and individual data value obtained from each sample.

## Supplementary Materials

### This PDF file includes:

Supplementary Materials and Methods Figs. S1 to S9 Table S1 to S7 Legends for data files S1 to S3 References

## Notes

View/request a protocol for this paper from *Bio-protocol*.
