# Supplementary material for: Microglia mediate neurocognitive deficits by eliminating C1q-tagged synapses in sepsis-associated encephalopathy
Source: Sci Adv. 2023 May 26;9(21):eabq7806. doi: 10.1126/sciadv.abq7806 (PMC10219600; doi:10.1126/sciadv.abq7806)
Supplement: Supplementary file 1 — Supplementary Materials and Methods Figs. S1 to S9 Table S1 to S7 Legends for data files S1 to S3 References [file sciadv.abq7806_sm.pdf]

Science Advances
AAAS

Supplementary Materials for

Microglia mediate neurocognitive deficits by eliminating C1q-tagged synapses in sepsis-associated encephalopathy

Ha-Yeun Chung *et al.*

Corresponding author: Christian Geis, christian.geis@med.uni-jena.de

Sci. Adv. **9**, eabq7806 (2023)
DOI: 10.1126/sciadv.abq7806

The PDF file includes:

Supplementary Materials and Methods
Figs. S1 to S9
Tables S1 to S7
Legends for data files S1 to S3
References

Other Supplementary Material for this manuscript includes the following:

Data files S1 to S3

Supplementary Material

Supplementary Material and Methods

Novel object recognition test

At day 10 and 30 after PCI, the novel object recognition test (NOR) was performed as previously described (*64*). A black open field box (40 cm), illuminated with 15 Lux via LEDs, was used for the experiment which were performed by a blinded experimenter. Objects were clearly distinguishable and different for each testing day: an iron bracket and an objective container (both day 10), a small plastic cylinder, and an iron eyebolt (both day 30). The objects were previously tested and no preferences for an individual object could be found. Additionally, each object was randomized to be used as familiar or unknown object. Between each trial, the box was carefully cleaned with 70% ethanol to ensure equal experimental conditions.

For habituation, mice were placed in the open field box for 10 minutes (5 min. for day 30) one day prior to the NOR. In the familiarization session on the following day, mice were allowed to explore two equal objects for maximum 10 minutes. Every touching of the object and facing an object in a distance of maximum 2 cm from the object was regarded as exploration behavior. Climbing onto the object was not regarded as exploration behavior. The familiarization phase was terminated by the investigator after mice had explored the two objects for cumulative 20 seconds. During the familiarization phase also the "time to reach criterion" was analyzed, meaning the time to reach 20 seconds at the objects to test any restrictions of locomotor or exploratory behavior. In the testing phase, six hours after the training phase, one object was replaced and mice were allowed to explore the familiar and unknown object. Exploration time was measured, and the session was completed after 20 seconds cumulative exploration behavior. Mice not exploring the objects for 20 seconds within 10 minutes in the familiarization or testing phase were excluded from the analysis.

Barnes Maze test

A circular white PVC platform (diameter 120 cm) was mounted on a rotatable stand, elevated 100 cm above floor level, and consistently illuminated by 2 light sources (900-1000 Lux). The maze comprised 40 pseudo-randomly distributed and equally sized holes (diameter each 5

cm), 39 holes were closed with black PVC. During the habituation (d31 after PCI) and testing phase (d32-37 after PCI) an escape box was mounted below one of the holes. Four different holes at the respective sides of the maze were used as escape holes. The location of the escape box was kept constant for each mouse but varied between mice (total of 4 possible target locations). Three different black-colored geometric shapes printed on paper were used as cues and placed at the three walls surrounding the maze. At the open side of the maze the examiner was regarded as the cue. Before starting each experiment, mice were acclimated to the testing room for at least 30 minutes and each mouse was placed in an individual holding cage after the experiment. After each trial the maze was cleaned with 70% ethanol to ensure comparable experimental conditions. In the habituation phase (d31), mice were placed in a transparent plastic located in the center of the maze. After 60 seconds the cylinder was lifted and mice were allowed to explore the maze for 60 seconds and then gently led to the escape box using the transparent cylinder. Mice were allowed to spend 2 minutes in the escape box before they were placed in individual holding cages. Each mouse underwent the habituation phase thrice with an inter-trial interval of 10 minutes. In the testing phase (d32-37), an opaque instead of the transparent plastic cylinder was used. The cylinder was lifted after 20 seconds and mice were videotaped and tracked (EthoVision) for maximum 4 minutes or until the mouse entered the escape box. Each mouse underwent the testing phase thrice with an inter-trial interval of 10 minutes. The mean latency until entry of the escape hole per day was calculated and used for further analysis (*54*).

Cell isolation

CD11b$^+$ primary microglia were isolated in all groups using MACS Technology (Miltenyi Biotec) according to manufacturer's instructions. Following preparation, brain samples were minced into small pieces and further dissociated enzymatically by Adult Brain Dissociation Kit (Miltenyi Biotec) and mechanically by gentle MACS Dissociator using program 37C_ABDK_01. A single-cell suspension was obtained after a separation by a 70µm cell strainer. Cell suspension was labelled magnetically using CD11b-MicroBeads (Miltenyi Biotec) and loaded onto a MACS column for positive separation. Cells were immediately lysed in 250µl QIAzol (Qiagen) for further transcriptomic analysis.

RNA purification

Total RNA extraction was carried out using QIAzol lysis reagent and RNeasy Mini Kit (Qiagen, Hilden, Germany) according to manufacturer instructions; quality control of isolated RNA was performed on a QIAxcel capillary electrophoresis system (Qiagen, Hilden, Germany). Integrity of RNA was proven by reporting the 8S/18S ratio and the RIS number (RNA Integrity Score) for analyzed samples. Quantification of total RNA and measuring the A260/A280 ratio and 260/230 nm were performed on a Nano-Drop spectrophotometer ND-2000 (Thermo Fisher Scientific, Schwerte, Germany).

RNA sequencing

Sequencing of RNA samples was performed using Illumina's next-generation sequencing methodology (*65*). In detail, total RNA (extraction see above) was quantified and quality checked using the Agilent 2100 Bioanalyzer Instrument in combination with RNA 6000 nano kit (both Agilent Technologies). Libraries were prepared from 500 ng (hippocampal samples) or 150 ng (microglial samples) of input material (total RNA) using NEBNext Ultra II Directional RNA Library Preparation Kit in combination with NEB Next Poly(A) mRNA Magnetic Isolation Module and NEBNext Multiplex Oligos for Illumina® (Index Primers Set 1/2/3/4) following the manufacturer's instructions (New England Biolabs) (*63*). Quantification and quality checked of libraries was done using the Agilent 2100 Bioanalyzer and DNA 7500 kit (hippocampal samples) or the Agilent 4200 Tapestation and D1000 screen tapes (microglial samples). Libraries were pooled and sequenced in five lanes (hippocampal samples) or two lanes (microglial samples) of the HiSeq 2500. System runs in 51 cycle/single-end/high-output mode. Sequence information was converted to FASTQ format using bcl2fastq v2.20.0.422.

Quantitative real-time PCR (qRT-PCR)

RNA, which was also used for RNA sequencing, was used to synthesize cDNA with the RevertAid First Strand cDNA Synthesis Kit (thermo scientific, #K1662). cDNA (5 ng), SYBR Green master mix (2x Brilliant III SYBR® Green QPCR Master Mix, # 600882-51 and primers (Table S1) were mixed and amplified with the following protocol in a Rotor gene Q (Qiagen/Corbett): Initial denaturation 95 °C for 3 min, 40 cycles of 95 °C for 10 sec and 60 °C for

15 sec and final denaturation 95 °C for 1 min. A melting curve was performed by gradually increasing from 55 °C to 95 °C in 80 steps. Data were analyzed with the software Rotor-Gene Q (QIAGEN, version 2.3.5) and Q-rex (QIAGEN, version 1.1) equipped with the Q-rex Gene Expression Plugin (version 2.0.0.4). Relative gene expression was calculated with the ddCT method with respect to two reference genes (*Hmbs* and *actB*) (*66*). According to the MIQE guidelines, primers were validated by defining the dynamic range and their efficiency as well as exclusion of primer dimer formation. Each experiment included three technical replicates, negative H2O controls and no reverse transcriptase controls

Table S1 Summary of primers used for qRT-PCR

Primer	Primer sequences (5'→ 3')	Concentration
**Csf1R**	fwd: AGCTCTCAGTACTTCAGGGCC<br>rev: GAGACATGACAGACATACAGGCC	0.5 µM
**TMEM119**	fwd: GACAGTCAGACCGTCTAGCG<br>rev: AGACAGGAGGAACCAGGGG	1 µM
**C1qC**	fwd: GGGAAGGACGGGCATGATGG<br>rev: CTTCTGACCCTTGGGTCCTCG	1 µM
**Hmbs**	fwd: GGATGTGCCTACCATACTACCTCC<br>rev: GGCAAGGTTTCCAGGGTCTTTCC	0.75 µM
**bAct**	fwd: GCTCTTTTCCAGCCTTCCTT<br>rev: CGGATGTCAACGTCACACTT	1 µM

Usage of software and bioinformatics resources for RNA sequencing analysis

Table S2 Summary of used software for RNA sequencing data

Software	Version	Reference
**R**	3.6.1	R Core Team, 2019
**R Studio**	1.1.383	R Studio Team, 2016
**Cytoscape**	3.8.0	(*67*)
**EnrichmentMap app**	3.2.1	(*68*)
**AutoAnnotate app**	1.3.2	(*69*)
**R Package**	**Version**	**Reference**
**readxl**	1.3.1	https://CRAN.r-project.org/package=readxl
**xlsx**	0.6.3	https://CRAN.r-project.org/package=xlsx
**VennDiagram**	1.6.20	https://CRAN.R-project.org/package=VennDiagram
**biomaRT**	2.40.5	https://CRAN.R-project.org/package=biomartr

**gridExtra**	2.3	https://CRAN.R-project.org/package=gridExtra
**gprofiler2**	0.1.8	https://CRAN.R-project.org/package=gprofiler2
**dplyr**	0.8.5	https://CRAN.R-project.org/package=dplyr
**ggpubr**	0.2.5	https://CRAN.R-project.org/package=ggpubr

FACS analysis

Transcardial perfusion with cold PBS (5ml/min, 4°C) for 2 min was performed to remove blood. Following brain removal, both hemispheres and the cerebellum were minced and subsequently digested enzymatically with collagenase, DNAse and trypsin-inhibitor for 45min at 37°C. Nondissociated brain tissue aggregates were removed using a 40 µm cell strainer. Cells were separated by standard density gradient centrifugation using Lymphoprep (Stemcell Technologies, Köln, Germany) and then centrifuged at 800g for 16min. Following centrifugation, the cell pellet was resuspended in FACS-buffer (PBS with 0.5% BSA, 2 mM EDTA and 0.1% sodium azide) for further analysis.

Fluorescence-labelled antibody staining for flow cytometric analysis of brain immune cells was performed in FACS buffer. Non-specific antibody binding was blocked by using IgG (Jackson, UK) for 10 min at 4°C prior antibody staining. Subsequently, cells were stained with fluorochrome-labeled antibody mixtures at 4 °C for 20 min. The following antibodies were used: APC anti-mouse CX3CR1 (2 µg/test, Cat# 149007, RRID:AB_2564491, Biolegend, San Diego, USA), APC/Cyanine7 anti-mouse F4/80 (1 µg/test, Cat# 123117, RRID:AB_893489, Biolegend, San Diego, USA), Brilliant Violet 605™ anti-mouse/human CD11b (1 µg/test, Cat# 101237, RRID:AB_11126744, Biolegend, San Diego, USA), VioGreen CD45, anti-mouse (1 µg/test,, Cat# 130-110-665, RRID:AB_2658225, Miltenyi Biotec, Bergisch-Gladbach, Germany). Upon staining, cells were analyzed using a FACS Canto Plus flow cytometer (BD Bioscience), and datawere analyzed using FlowJo X Software (BD Bioscience). Representative gating strategy for $CD45^{low}CD11b^{+}CX3CR1^{+}$ microglia, $CD45^{high}CD11b^{+}CX3CR1^{+}F4/80^{+}$ macrophages and $CD45^{high}CD11b^{+}CX3CR1^{+}F4/80^{-}$ monocytes is presented in Fig. S1B.

Capillary western immunoassay

Mouse hippocampi were homogenized in lysis buffer (0.32 M Sucrose, 4 mM Tris-HCL (pH = 7.4), 1 mM EDTA, 0.25 mM DTT) with additional ultrasound sonication (10 %, 5x pulses). The protein concentration was determined using Bradford assay (BioRad Quick Start Bradford 1x

Dye Reagent, #5000205) prior to protein analysis by capillary western immunoassay (WesTM, ProteinSimple) according to the user manual. For each target, the antibody dilution and dynamic range were determined independently to assure quantitative measurements (Table S3). The antibody incubation time of primary as well as of secondary antibodies was 30 min. Outlier were excluded after performing Grubbs' Test (1 in SHAM/AIN Synaptophysin d3; 1 in PCI/AIN Synaptophysin d3) (*54*).

Table S3 Summary of antibodies used for capillary western immunoassay

1°AB	Host	Established dilution	Protein Load	AB Catalogue #
anti-PSD-95	rabbit	1:150	0.02 µg/µl	CST# 3450<br>RRID:AB_2292883
anti-SYP	rabbit	1:50	0.4 µg/µl	ab52636<br>RRID:AB_882786
anti-GAPDH	rabbit	1:25	0.02 µg/µl	CST#2118<br>RRID:AB_561053
anti-GAPDH-HRP	mouse	1:5	0.4 µg/µl	MA5-15738<br>RRID:AB_10977387

Hippocampal patch-clamp recordings

9-13 week-old mice were deeply anesthetized with isoflurane, then decapitated, after which the brain was quickly removed into ice-cold paCSF (containing in mM: 95 N-Methyl-D-Glucamine, 30 NaHCO3, 2.5 KCl, 1.25 NaH2PO4, 10 MgSO4, 0.5 CaCl2, 20 HEPES, 25 glucose, 2 thiourea, 5 Na-ascorbate, 3 Na-pyruvate, 12 N-acetylcysteine, adjusted to pH 7.3 and 300-310 mOsmol, saturated with carbogen). Transversal 350µm-thick hippocampal slices were prepared with a vibratome (VT1200S; Leica, Wetzlar, Germany) in ice-cold paCSF and were transferred for warm recovery in paCSF at 32°C for 12 minutes. Thereafter, slices were left to recover for at least one hour at room temperature in aCSF+ (containing in mM: 125 NaCl, 25 NaHCO3, 2.5 KCl, 1.25 NaH2PO4, 1 MgCl2, 2 CaCl2, 25 glucose, 2 thiourea, 5 Na-ascorbate, 3 Na-pyruvate, 12 N-acetylcysteine, adjusted to pH 7.3 and an osmolarity of 300–310 mOsmol, and saturated with carbogen). CA1 pyramidal neurons were visually identified using a microscope (Examiner.Z1, Zeiss, Jena, Germany) equipped with differential interference contrast optics. Patch pipettes were pulled using a P-87 horizontal pipette puller (Sutter Instruments, Novato, CA, USA) from thick-

walled borosilicate glass (0.86x1.50, Science Products, Kamenz, Germany) and had a resistance of 2.5-4.5 MΩ when filled with an intracellular solution (containing in mM: 135 CsMeSO4, 3 CsCl, 10 HEPES, 0.2 EGTA, 0.1 spermine, 2 QX-314-Br (Tocris, Bristol, UK), 2 Mg-ATP, 0.3 Na2-GTP, 10 phosphocreatine, pH 7.25, osmolarity 280 mOsmol). Biocytin 4mg/ml (Tocris, Bristol, UK) was freshly added to the intracellular solution on the day of recording. The reversal potential of inhibition was experimentally measured at -70 mV, and the experimentally determined liquid junction potential (8 mV) was corrected on-line. Whole-cell voltage clamp recordings from CA1 pyramidal neurons were performed using a HEKA EPC-10 patch-clamp amplifier at a sampling rate of 20 kHz after a low-pass filtering of 2.9 kHz using the amplifier's Bessel filter. Recordings were performed in aCSF (containing in mM 125 NaCl, 2.5 KCl, 25 NaHCO3, 1.25 NaH2PO4, 1 MgCl2, 2CaCl2, saturated with carbogen) at room temperature and at a flow rate of 2 ml/min (Ismatec, Wertheim, Germany). Series resistance (<25 mΩ, 70-80 % compensated) was monitored during recordings and cells with a change over 20 % from baseline were discarded. Miniature excitatory postsynaptic currents (mEPSC) were recorded for 5 minutes in the presence of 1 µM TTX (Tocris, Bristol, UK) at a holding potential of -70 mV. Analysis of mEPSCs was performed with MiniAnalysis 6.0 (Synaptosoft, Decature, GA, USA), detected events were reviewed and manually confirmed by a blinded user (*54*). Chemicals were purchased from Sigma-Aldrich, St. Louis, MO, USA, unless stated otherwise.

Biocytin staining and morphologic analysis

Slices after patch-clamp recordings were transferred in 4% PFA and fixed overnight at 4°C. After washing in TBS, slices were incubated for two hours in TBS plus (TBS, 5 % donkey serum, 0.3 % Triton-X-100) and then incubated overnight in 1:500 Streptavidin-Cy3 (Sigma, # S6402) in TBS plus before embedding in Entellan (Merck, Darmstadt, Germany). Biocytin filled neurons were captured on a confocal scanning microscope (LSM 710, Zeiss, Jena, Germany). Three regions of a filled neuron were imaged. Morphologic analysis was performed in Imaris7 (Bitplane, Belfast, UK) and the mean result per filled neuron was used for further analysis. The researchers performing biocytin staining, imaging and consecutive analyses were blinded to the experimental treatment of the animals.

Measurement of serum neurofilament, UCH-L1 and tau protein

NfL assay by Quanterix HD-X was used to measure neurofilament light chain (Nfl) levels. All available serum samples were analyzed for concentrations of Nfl, glial fibrillary acidic protein (GFAP), ubiquitin carboxy-terminal hydrolase L1 (UCH-L1), and tau protein in pg/ml using a single molecule array technique with a commercially available 4-plex kit (Neurology 4-plex kit, Quanterix) on a HD-X platform. Results were quantified using calibration curves with known standards assayed in the same run. Assays were done according to the manufacturers protocols except for analyzing singlicates (due to limited amount of serum) instead of duplicates after determining inter- and intra-assay coefficient of variance for Nfl/GFAP high and low controls to be below 10 %. The analysis was performed by blinded experimenters.

We performed inter-assay, intra-assay comparisons and precision measurements as well as inter-laboratory comparisons of Nfl and GFAP (Table S4) and identified a very low coefficient of variance in these parameters and excellent inter-laboratory correlations (Pearson correlation coefficient 0,9986) as described elsewhere (*70*).

Table S4 Intra- and inter-assay precision in low and high NfL concentration samples (from (*70*)).

Sample	Plex	Mean (pg/ml)	Intra-Assay CV (%)*	Inter-Assay CV (%)*
Low	NfL	8,67	6,43	8,20
	GFAP	39,84	4,13	4,87
High	NfL	142,45	1,96	5,83
	GFAP	107,85	4,82	5,86

NfL - neurofilament light; GFAP - glial fibrillary acidic protein; CV - coefficient of variance

ELISA

The blocking functionality of the C1q blocking antibody was validated by ELISA assays targeting C3b, a protein that finally tags cells for microglial phagocytosis downstream of C1q-mediated classical complement pathway activation (*71*). To that end, human complement active serum (normal human serum, NHS) was collected with hirudin monovettes from five healthy donors (age 22-32 years, female and male). The samples were centrifuged at 10.000 rpm for 10 min at 4°C, pooled and stored at -20°C for up to 3 month.

Plates (96 well, NUNC, Thermofisher) were coated with IgM (concentration 0.2-2µg/µl, abcam ab91117) in 100 µl coating buffer (Merck C3041) overnight at 4°C for classical complement activation or C3b (2.5-0.00016 µg/ml, Merck 204860-250UG) for standard curve determination. The wells were washed three times with wash buffer (TBS + 0.1% Tween) and blocked with blocking buffer (wash buffer, 3 % milk powder, 1% BSA). C1q blocking antibody (6.1 µl, concentration 0.41-0.0041 µg/µl) was pre-incubated with 1 µl NHS or C1q-depleted serum (TecoMedical A300C) or PBS for 15 min at 37°C and finally diluted with 90 µl GVB++ buffer (TecoMedical B100). Then, the probes were applied to the 96-well plate and incubated for 5 min at 37°C. Complement activity was stopped by 100 µl GVBE buffer (TecoMedical B104) and the wells were washed 3 times before incubation with C3b antibody solution (dilution 1:1.500 in 50 µl blocking buffer/well, TecoMedical A213c) for 1 h at room temperature. After 3 additional washing steps, HRP - labeled secondary antibody (dilution 1:2.000 in 50 µl blocking buffer/well, abcam ab6741, RRID:AB_955424) was incubated for 30 min at room temperature. Finally, 100 µl TMB substrate solution (Serva 37068.01) were added for 15 min, the reaction stopped with 0.5 M sulfuric acid and the absorption at 450 nm and 570 nm measured. Standard curves and samples were measured in technical duplicates and analyzed based on their average.

Stereotactic intrahippocampal injection of antibodies

C1q blocking antibody (4.5mg/ml in PBS) and control antibody without specific anti-neuronal reactivity (purified patient IgG fractions, 4.5 mg/ml) were injected at 3 injection sites per hemisphere (Table S5) into the hippocampus 2 days prior to sepsis induction.

Table S5 Coordinates for antibody injection sites into the hippocampus referring to bregma in mm

Hemisphere	Anterior-posterior	Medial-laterale	Dorso-ventrale
Left hemisphere (C1q Ab)	-2.1	-1.4	-1.4
	-2.1	-1.4	-2.0
	-2.8	-2.8	-2.4
Right hemisphere (control Ab)	-2.1	1.4	-1.4
	-2.1	1.4	-2.0
	-2.8	2.8	-2.4

Analgesia was ensured by pre – (1h) and post-operative (24h) application of meloxicam (1 mg/kg). During anesthesia with 2.5 % isoflurane in oxygen, the body core temperature was monitored by a rectal temperature probe and adjusted to 37°C by a heating pad with feedback control (FHC 40-90-8D). Corneal drying was prevented by eye ointment. Stereotactic

intrahippocampal injections were performed as established (*72*). The head was shaved and fixed with a stereotactic apparatus (Stoelting, 51500 D). After a small midline incision to expose the skull, four holes (diameter 1 mm) were carefully drilled into the skull by a dentist driller (HM1 005, #500 104 001 001, Meisinger, Germany) under visual inspection with the aid of a stereo microscope (Olympus SZ60, Objective 100 AL 0.5x WD186). At each injection site, 1 µl of antibody was injected with an injection speed of 4 nl/s using a nanoliter injector (Nanoliter 2000+ SYS-Micro4 Controller; WPI). Long taper injection glass pipettes (glass capillaries, WPI #4878) were pulled with a micropipette puller (P-1000, Sutter) and cut with fine scissors to a diameter of several micrometers. After the injection finished, the injection pipette was carefully removed after a latency of 1-2 minutes and the surgical incision was closed using 2-3 michel suture clips (#BN507R, BBraun, Germany). Animals were monitored until they completely recovered from anesthesia.

Immunohistochemistry (mouse tissue)

To obtain mouse brain slices for immunohistochemistry, mice were deeply anesthetized with isoflurane and perfused with PBS after cardiac puncture. After preparation, brains were fixated for 24 h in 4% PFA, and dehydrated for 24 h in 10% and 24 h in 30% sucrose solution. Thereafter, free-floating serial sections (16 µm and 40 µm respectively) were prepared. Slices were blocked with BB 2 (3% serum, 2% milk powder and 0.1% Triton X-100 in TRIS buffered saline [TBS]) for 30 min. Thereafter, slices were incubated overnight at 4°C with primary antibody iba1 (rb, 1:500, Cat# 019-19741, RRID:AB_839504, Wako Pure Chemical Industries, Japan; gp, 1:100, Cat# 234004, RRID:AB_2493179, SySy, Göttingen, Germany), C1q (rb, 0.5 µg/µl, Cat# ab182451, RRID:AB_2732849, abcam, Cambridge, UK,), Homer1 (ch, 1:500, Cat# 160 006, RRID:AB_2631222, SySy, Göttingen, Germany), or CD68 (ms, 1:500, Cat# ab955, RRID:AB_307338, abcam, Cambridge, UK). Following washing steps in TBS, slices were incubated for 2h with secondary antibody (donkey@ms IgG (H+L), CF568, 1:500, Cat#20105, RRID:AB_10557030, Biotium, USA; donkey@rb, CF568, 1:500, Cat#20098, RRID:AB_10557118, Biotium, USA; donkey@rb, AF488, 1:500, Cat#A21206, RRID:AB_2535792, Thermofisher, Waltham, USA; donkey@rb, rhodamin, 1:500, Cat#711-297-003, RRID:AB_2340615, Jackson Laboratory Immuno Research, West Grove, USA; donkey@ms, AF647, 1:500, Cat#715-605-151, RRID:AB_2340863, Jackson Laboratory Immuno

Research, West Grove, USA; goat@ch IgY (H+L), AF488, 1:200, Cat# A-11039, RRID:AB_142924, Invitrogen, Waltham, USA; goat@gp, AF 405, 1:200, Cat# ab175678, RRID:AB_2827755, abcam, Cambridge, UK). After additional washing in TBS, slices were transferred to object slides with 0.5% gelatin, dried, stained for 5 min in DAPI (Sigma Aldrich, CAT# D9542, 1mg/ml) solution, washed in PBS and mounted with Fluoromount G (Southern Biotech, Birmingham, AL, USA).

Imaging and Image analysis (mouse tissue)

Immunostained brain slices were imaged with confocal laser scanning microscopes (Zeiss LSM 710; Zeiss LSM 900 in part with Airyscan mode) and Zeiss Elyra 7 for super-resolved lattice-structured illumination microscopy (lattice-SIM). The researchers performing staining, imaging and consecutive analyses were blinded with respect to the treatment of samples.

To quantify hippocampal microglia stained for iba1 20x (NA = 0.8) magnified z-stacks with a z-interval of 3 µm and 4 pixel-wise plane scan intensity averages for each color channel were collected (3 image stacks/mouse; 4 mice/group). Cells were quantified using Fiji.

Full thickness 63x (NA = 1.4) z-stack images of the hippocampal CA1 region were collected from sections immunostained for iba1 and DAPI (10 z-stacks per mouse, 4 mice/group,). z-stacks were collected at 1024 x 1024 resolution with 4 pixel-wise plane scan intensity averages for each color channel and 0.7 µm z-step size. Images were median filtered via a custom written script and subsequently analyzed using Imaris (version 9.1.2.; Oxford instruments).

To analyze CD68 and iba1 colocalization, z-stack images of the CA1 region were acquired on LSM900 (40x objective, NA = 0.95) with a z-interval of 10.26 µm, an image resolution of 1024 x 1024, and 8 image pixel-wise plane scan intensity averages (2 image stacks/mouse; 5 mice/group). The colocalized CD68+ signal volume resulting from the structural overlap of iba1 and CD68 was normalized to the number of iba1+ microglia. Colocalized CD68+ volume was determined using Imaris (version 9.1.2.; Oxford instruments). The microglia number was manually assessed.

To study microglial synapse engulfment, Airyscan imaging was performed (LSM900, 63x oil objective, NA = 1.4). z-stack images of 16 µm thick sections stained for iba1, Homer1 and DAPI (2-3 image stacks/mouse, 5 mice/group) were acquired in the CA1 region with a 1490 x 1490 image resolution and 4 pixel-wise plane scan intensity averages for each color channel and

0.3 µm z-step size. Images were background subtracted (threshold: maximum of the histogram of each fluorophore) and analyzed using the surface function in Imaris (threshold: automatic) for the two fluorescence channels iba1 and Homer1. The colocalization function and an implemented MatLab code in Imaris was used to assess the colocalization of the iba1 and Homer1 signal. Results were filtered with a threshold of 0.1 µm³.

C1q intensity analysis was performed in 40µm thick sections. Staining and imaging, respectively were performed at the same day. Hippocampal tile scans were collected (10x objective, NA = 0.3; d3: 7-9 mice/group, 3 images/mouse; d10: 6-8 mice/group, 3 images/mouse), and fluorescence intensity was assessed using Fiji. Results were normalized to the control group.

For C1q-Homer1 colocalization analysis 16 µm thick section were stained using C1q, Homer1 and DAPI. Images were acquired on LSM900 (63x oil objective; NA = 1.4) using Airyscan mode (d3 and d10: 5 images/mouse, 5 mice/group). The 2D scan was performed with a 1466 x 1466 image resolution and with 8 pixel-wise plane scan intensity averages for each color channel. Colocalization analysis was performed with the spot function in Imaris (threshold: automatic). Hereby, surfaces with a diameter of 0.2 µm were established for both fluorophore signals. Finally, the established surfaces were colocalized using a MatLab code with a distance between the two intensity maxima of the spots of ≤ 0.25 µm. This value was determined using the intensity profile along the plotted line (Fig. 6C) and by the distance between the two intensity maxima.

For representative imaging of hippocampal C1q-labeled synapses engulfed in lysosomal microglial compartments, images were acquired on LSM900 (63x oil objective, NA = 1.4) using Airyscan mode. For this purpose, murine brain sections at d10 after PCI were stained for iba1, Homer1, CD68 as well as C1q. z-stacks were acquired with a 2028 x 2028 image resolution and with 8 pixel-wise plane scan intensity averages for each color channel and a 7.8 µm z-interval. To visualize C1q-dependent synapse elimination, representative microglia were selected and reconstructed in Imaris. For this purpose, the surface, the spot as well as the colocalization function was used for 3D reconstruction. C1q labeling of postsynaptic Homer1 was visualized using the "spots" function.

To validate specific binding of the C1q blocking antibody and to evaluate potential antibody diffusion after injection to the contralateral hemisphere, 16 µM thick tissue slices were

stained against C1q (in addition with a secondary antibody targeting mouse IgGs). Images were acquired with LSM900 (63x oil objective, NA = 1.4) using the Airyscan mode.

To quantify synaptic spots 10µm thick cryo slices stained with Homer1 and DAPI were imaged at two different locations in the CA1 region (8 images/mouse, 5-6 mice/group) on a Zeiss Elyra 7 lattice-SIM (63x oil objective, NA = 1.4). Images were acquired using the SIM function with a 1024 x 1024 image resolution. Scans were processed using Zen Software and Imaris. Synapse quantification was performed with Imaris (surface function)

Immunohistochemistry (human tissue)

Three-micrometer thick sections from formalin-fixed paraffin-embedded autopsy brain tissue blocks were cut and processed for immunohistochemistry using standard routine procedures. Tissue sections were stained with hematoxylin and eosin (H&E) and additional antibodies against CD68 (1:4000; Cat# M081401-2, RRID:AB_2750584, Agilent, USA), C1q (rb, 1:200, Cat# PA5-106648, RRID:AB_2854316, invitrogen, Waltham, USA) and iba1 (1:1000; Cat# 019-19741, RRID:AB_839504, Wako Pure Chemical Industries, Japan) in an Agilent autostainer.

For additional immunofluorescence stainings, sections were deparaffinized prior to staining (NeoClear (Merck, Germany), 100% ethanol, 90% ethanol, 70% ethanol, 50% ethanol each 2x 5min, aqua dest 5min, TBS 5min). To unmask antigen, slides were covered with citrate buffer (10mM, ph6) and microwaved at 800W (5 min). After cooling down and a washing step (TBS-T), blocking solution (5% BSA) was added (2h, room temperature). Afterwards, slides were incubated with blocking solution and iba1 (rb, 1:500, Cat# 019-19741, RRID:AB_839504, Wako Pure Chemical Industries, Japan) overnight (4°C). After washing steps, slices were incubated with secondary antibody (donkey@rb, CF568, 1:500, Cat#20098, RRID:AB_10557118, Biotium, USA) for 2h at room temperature. After additional washing steps, DAPI (Sigma Aldrich, CAT# D9542, 1mg/ml) was added. After washing, slices were mounted with Fluoromount G (Southern Biotech, Birmingham, AL, USA).

Imaging and Image analysis (human tissue)

Two (CD68 and iba1 stainings) and three (C1q stainings), respectively, hippocampal images per patient were taken in the CA1 region (20x objective, Axioplan 2, Zeiss, Germany; DMIL LED Fluo, Leica, Wetzlar, Germany). Specifically stained cells and all H&E positive cells

(except for neurons of the dentate gyrus) were subsequently counted manually using Fiji to achieve normalization.

For analysis of human microglia of the immunofluorescence staining, representative z-stack images were acquired (LSM 900, Zeiss, Germany; 63x oil objective, NA = 1.4) in the CA1 region. Z-stacks were imaged with a 1024 x 1024 resolution and 15 µm z-interval. Subsequently, a manual analysis of the soma size was performed in Imaris (version 9.1.2.; Oxford instruments) by an experimenter blinded to both groups: The maximal longitudinal (a) and cross section (b) of the soma was measured. Soma size was calculated using the formula for calculating the area of an ellipse. 2D soma size = ½ a x ½ b x π.

Supplementary figures and legends
Fig. S1

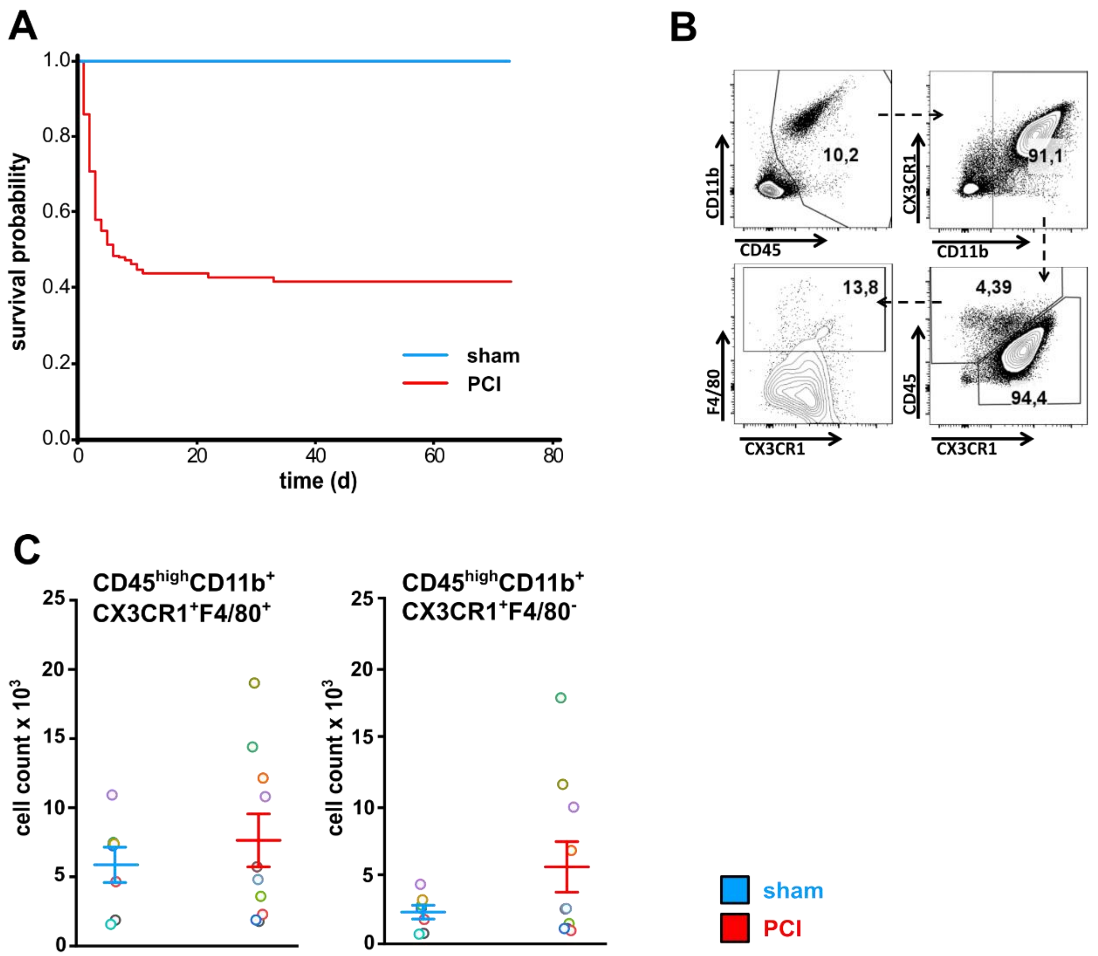

Fig. S1: Experimental sepsis results in 40-50% long-time survival and does not affect the number of brain-associated macrophages and monocytes

(**A**) Kaplan-Meier survival analysis of sham and PCI treated animals (Log-Rank-Test: $p < 0.001$). (**B**) Representative FACS plots and (**C**) flow cytometry analysis of $CD45^{high}CD11b^{+}CX3CR1^{+}F4/80^{+}$ macrophages and $CD45^{high}CD11b^{+}CX3CR1^{+}F4/80^{-}$ monocytes at day 3 following sepsis induction (sham: n = 7, PCI: n = 10; two-tailed Student's t-test).

Data are presented as mean ± SEM. Each circle represents one mouse.

Fig. S2

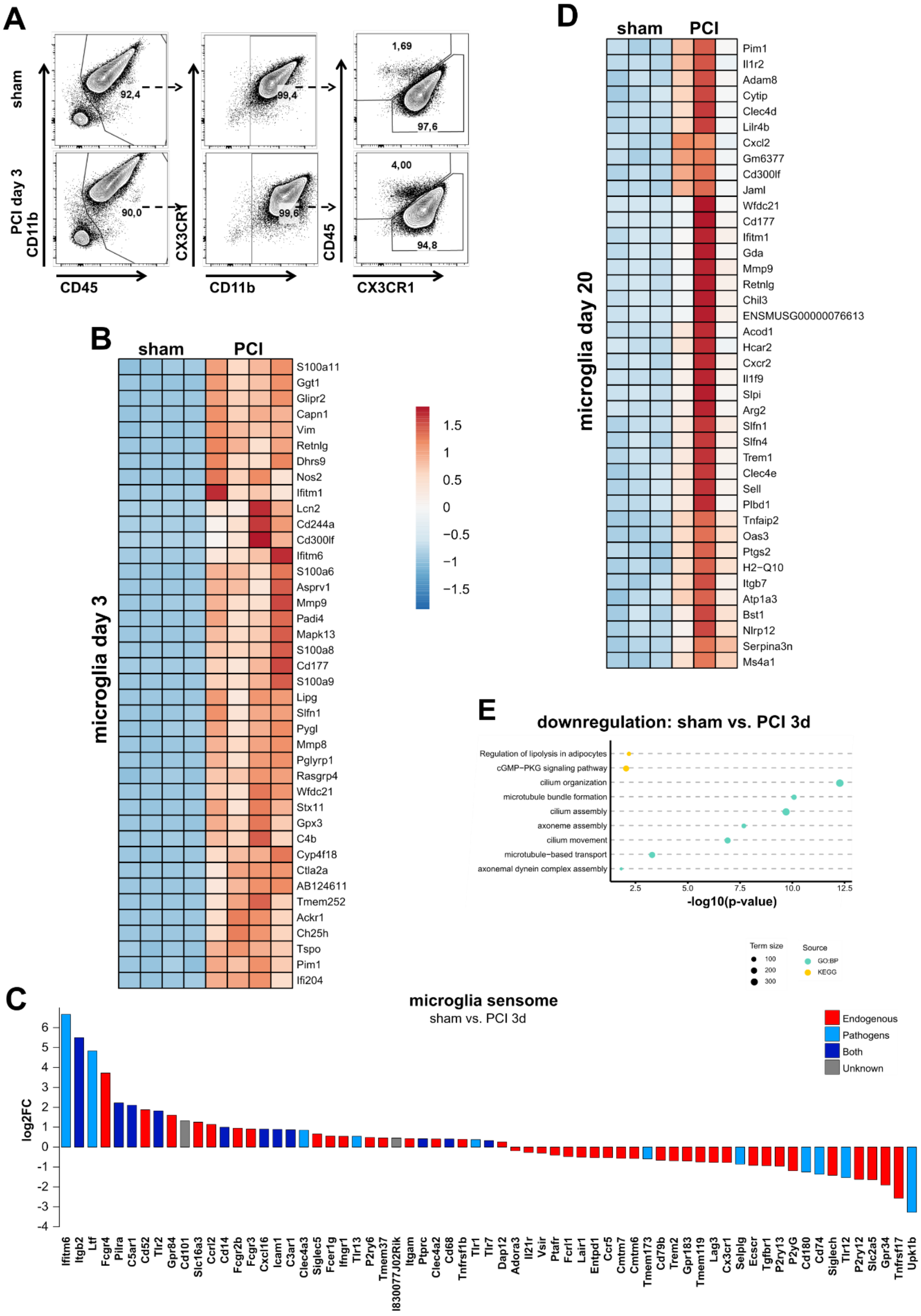

A
sham
PCI day 3
CD11b
CD45
92,4
90,0
CX3CR1
CD11b
99,4
99,6
CD45
CX3CR1
1,69
97,6
4,00
94,8
B
sham
PCI
microglia day 3
S100a11
Ggt1
Glipr2
Capn1
Vim
Retnlg
Dhrs9
Nos2
Ifitm1
Lcn2
Cd244a
Cd300lf
Ifitm6
S100a6
Asprv1
Mmp9
Padi4
Mapk13
S100a8
Cd177
S100a9
Lipg
Slfn1
Pygl
Mmp8
Pglyrp1
Rasgrp4
Wfdc21
Stx11
Gpx3
C4b
Cyp4f18
Ctla2a
AB124611
Tmem252
Ackr1
Ch25h
Tspo
Pim1
Ifi204
1.5
1
0.5
0
−0.5
−1
−1.5
D
sham
PCI
microglia day 20
Pim1
Il1r2
Adam8
Cytip
Clec4d
Lilr4b
Cxcl2
Gm6377
Cd300lf
Jaml
Wfdc21
Cd177
Ifitm1
Gda
Mmp9
Retnlg
Chil3
ENSMUSG00000076613
Acod1
Hcar2
Cxcr2
Il1f9
Slpi
Arg2
Slfn1
Slfn4
Trem1
Clec4e
Sell
Plbd1
Tnfaip2
Oas3
Ptgs2
H2−Q10
Itgb7
Atp1a3
Bst1
Nlrp12
Serpina3n
Ms4a1
E
downregulation: sham vs. PCI 3d
Regulation of lipolysis in adipocytes
cGMP−PKG signaling pathway
cilium organization
microtubule bundle formation
cilium assembly
axoneme assembly
cilium movement
microtubule−based transport
axonemal dynein complex assembly
2.5
5.0
7.5
10.0
12.5
-log10(p-value)
Term size
100
200
300
Source
GO:BP
KEGG
C
microglia sensome
sham vs. PCI 3d
Endogenous
Pathogens
Both
Unknown
log2FC
6
5
4
3
2
1
0
-1
-2
-3
-4
Ifitm6
Itgb2
Ltf
Fcgr4
Pilra
C5ar1
Cd52
Tlr2
Gpr84
Cd101
Slc16a3
Ccrl2
Cd14
Fcgr2b
Fcgr3
Cxcl16
Icam1
C3ar1
Clec4a3
Siglec5
Fcer1g
Ifngr1
Tlr13
P2ry6
Tmem37
I830077J02Rik
Itgam
Ptprc
Clec4a2
Cd68
Tnfrsf1b
Tlr1
Tlr7
Dap12
Adora3
Il21r
Vsir
Ptafr
Fcrl1
Lair1
Entpd1
Ccr5
Cmtm7
Cmtm6
Tmem173
Cd79b
Trem2
Gpr183
Tmem119
Lag3
Cx3cr1
Selplg
Escr
Tgfbr1
P2ry13
P2yG
Cd180
Cd74
Siglech
Tlr12
P2ry12
Slc2a5
Gpr34
Tnfrsf17
Upk1b

Fig. S2: Transcriptome analysis shows acute and prolonged activation of microglia at day 3 and day 20 after sepsis

(**A**) Flowcytometric analysis of $CD45^{low}CD11b^{+}CX3CR1^{+}$ microglia among MACS isolated CD11b positive cells. (**B** and **D**) Heatmap of top 40 DEGs of isolated microglia at day 3 (n = 4, padj < 0.05) and day 20 (n = 3, padj < 0.05) in PCI and sham treated animals. The color scale represents the gene-wise z-score calculated from normalized gene expression levels. (**C**) DEGs related to microglia sensome of RNA sequencing data at day 3 following PCI (n = 4/group). (**E**) Enrichment analysis of downregulated GO-BP and KEGG comparing PCI vs. sham at day 3 (n = 4, padj < 0.05) following sepsis induction.

Fig. S3

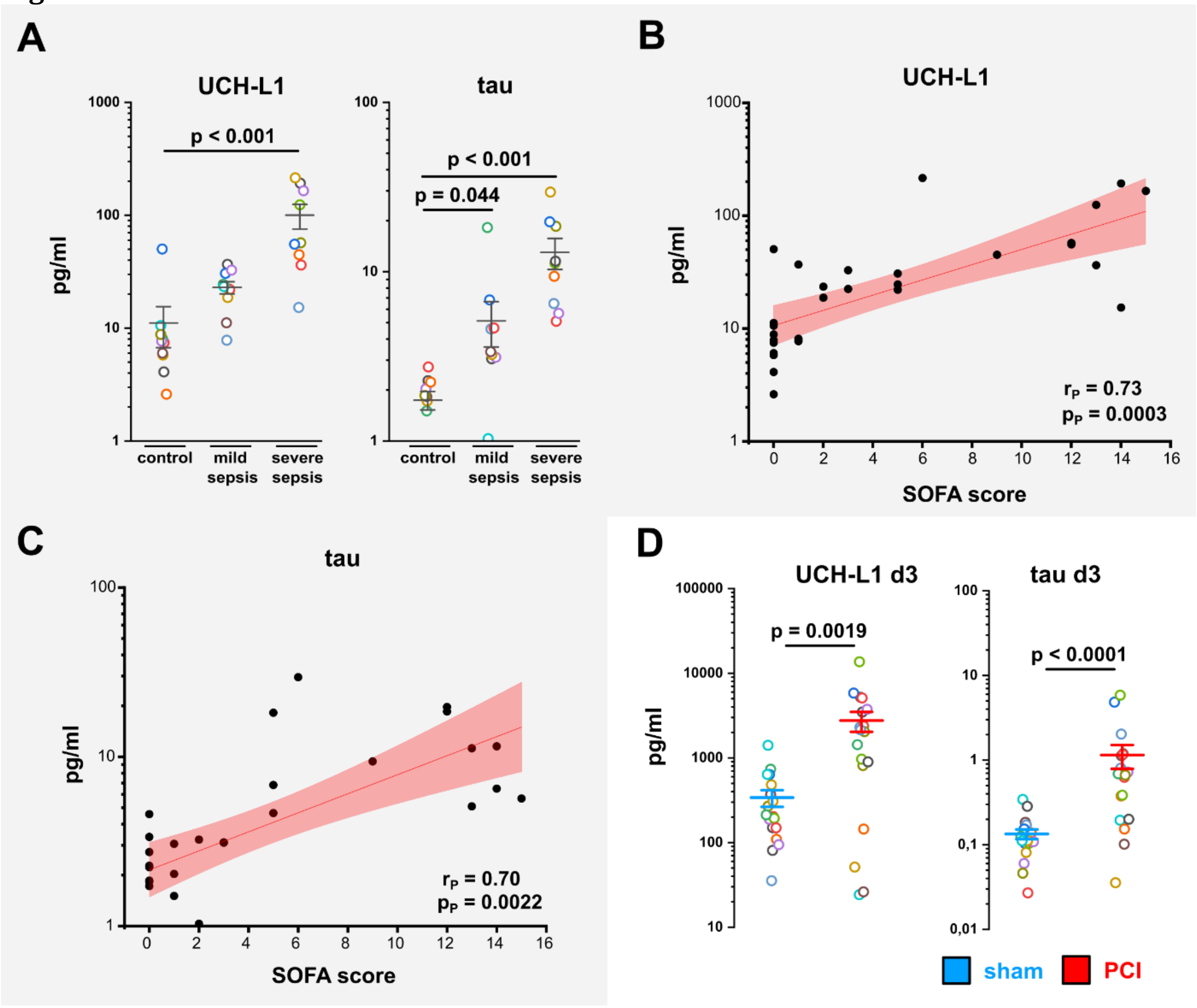

A
UCH-L1
tau
pg/ml
p < 0.001
p < 0.001
p = 0.044
control
mild sepsis
severe sepsis
B
UCH-L1
pg/ml
SOFA score
rP = 0.73
pP = 0.0003
C
tau
pg/ml
SOFA score
rP = 0.70
pP = 0.0022
D
UCH-L1 d3
tau d3
pg/ml
p = 0.0019
p < 0.0001
sham
PCI

Fig. S3: Blood-based biomarkers indicate neuronal injury in human and mice after sepsis

(**A**) Measurement of UCH-L1 and tau in control patients and patients with mild or severe sepsis (n = 10/group; Kruskal-Wallis ANOVA with Dunn's method for multiple comparisons test). (**B**) Correlation analysis between serum UCH-L1 and SOFA score (n = 30; Pearson correlation). (**C**) Correlation analysis between serum tau and SOFA score (n = 30; Pearson correlation). (**D**) Measurement of UCH-L1 and tau in serum sham and PCI-treated animals at day 3 (sham: n = 21; PCI: n = 18; two-tailed Student's t-test) following sepsis induction.

Data are presented as mean ± SEM. Grey shading (in A, B, C) indicates human data.

Fig. S4

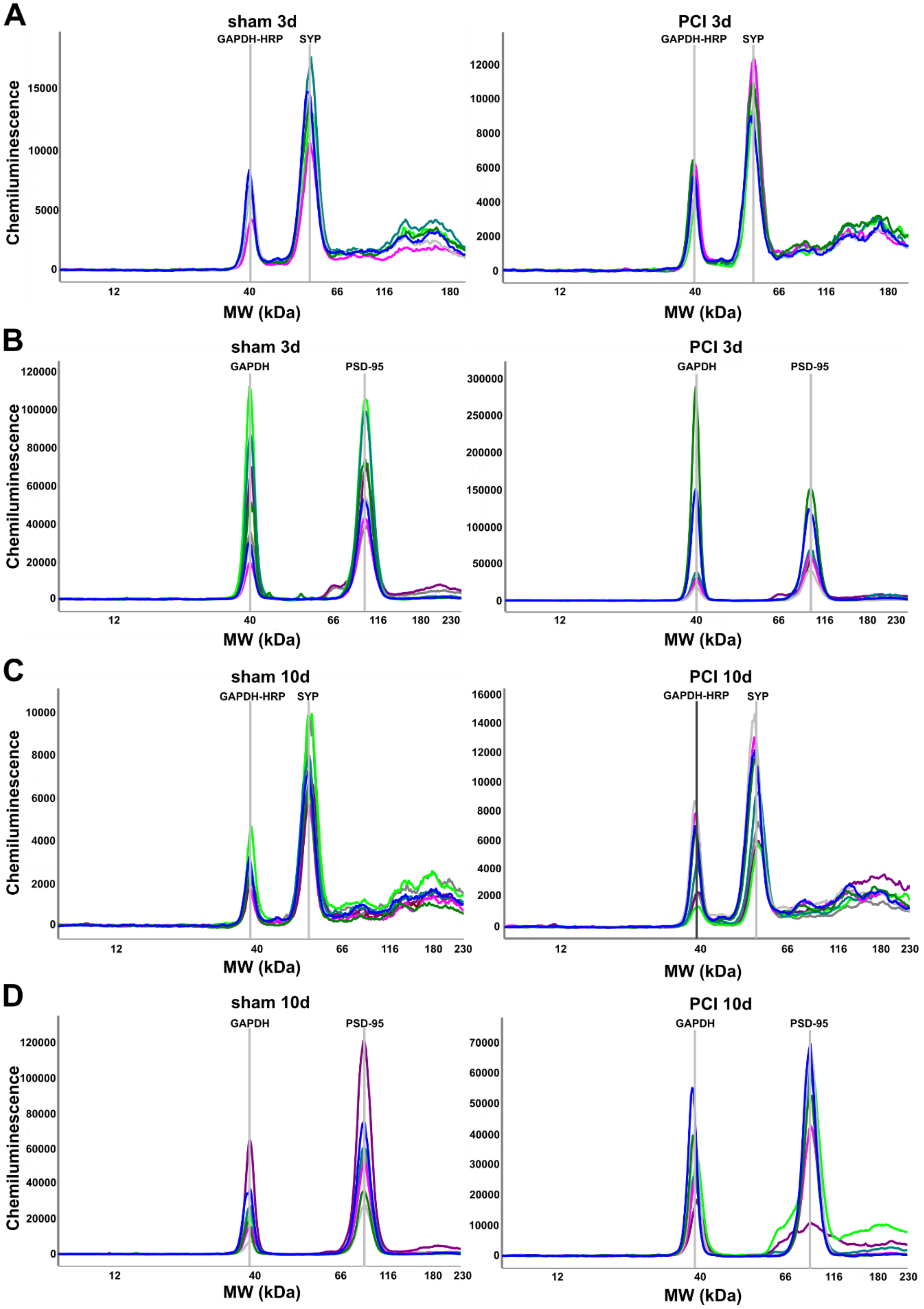
A
sham 3d
GAPDH-HRP
SYP
Chemiluminescence
15000
10000
5000
0
12
40
66
116
180
MW (kDa)
PCI 3d
GAPDH-HRP
SYP
12000
10000
8000
6000
4000
2000
0
12
40
66
116
180
MW (kDa)
B
sham 3d
GAPDH
PSD-95
120000
100000
80000
60000
40000
20000
0
12
40
66
116
180
230
MW (kDa)
PCI 3d
GAPDH
PSD-95
300000
250000
200000
150000
100000
50000
0
12
40
66
116
180
230
MW (kDa)
C
sham 10d
GAPDH-HRP
SYP
10000
8000
6000
4000
2000
0
12
40
66
116
180
230
MW (kDa)
PCI 10d
GAPDH-HRP
SYP
16000
14000
12000
10000
8000
6000
4000
2000
0
12
40
66
116
180
230
MW (kDa)
D
sham 10d
GAPDH
PSD-95
120000
100000
80000
60000
40000
20000
0
12
40
66
116
180
230
MW (kDa)
PCI 10d
GAPDH
PSD-95
70000
60000
50000
40000
30000
20000
10000
0
12
40
66
116
180
230
MW (kDa)

Fig. S4: Analysis of PSD-95 and synaptophysin indicates synaptic injury after sepsis

Capillary western immunoassay (WES) results of sham and PCI animals are shown as electropherograms. WES was performed targeting synaptophysin (SYP) and PSD-95 **(A-B)** at day 3 (sham: n = 7, PCI: n = 7) and **(C-D)** day 10 (sham: n = 8, PCI: n = 8) following sepsis induction.

Fig. S5

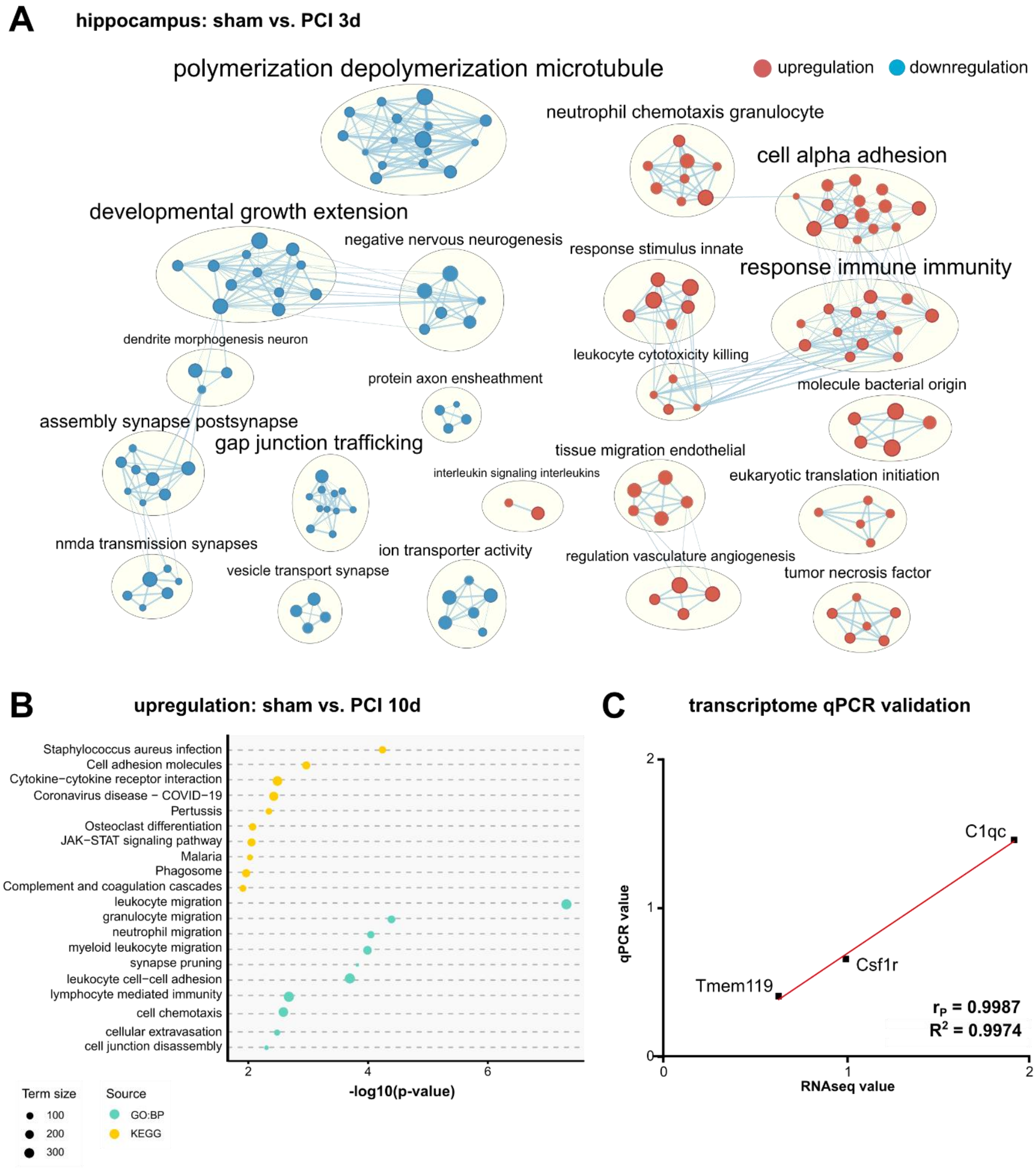
A
hippocampus: sham vs. PCI 3d
upregulation
downregulation
polymerization depolymerization microtubule
neutrophil chemotaxis granulocyte
cell alpha adhesion
developmental growth extension
negative nervous neurogenesis
response stimulus innate
response immune immunity
dendrite morphogenesis neuron
leukocyte cytotoxicity killing
protein axon ensheathment
molecule bacterial origin
assembly synapse postsynapse
gap junction trafficking
tissue migration endothelial
interleukin signaling interleukins
eukaryotic translation initiation
nmda transmission synapses
ion transporter activity
regulation vasculature angiogenesis
vesicle transport synapse
tumor necrosis factor
B
upregulation: sham vs. PCI 10d
Staphylococcus aureus infection
Cell adhesion molecules
Cytokine−cytokine receptor interaction
Coronavirus disease − COVID−19
Pertussis
Osteoclast differentiation
JAK−STAT signaling pathway
Malaria
Phagosome
Complement and coagulation cascades
leukocyte migration
granulocyte migration
neutrophil migration
myeloid leukocyte migration
synapse pruning
leukocyte cell−cell adhesion
lymphocyte mediated immunity
cell chemotaxis
cellular extravasation
cell junction disassembly
2
4
6
-log10(p-value)
Term size
100
200
300
Source
GO:BP
KEGG
C
transcriptome qPCR validation
qPCR value
RNAseq value
0
1
2
C1qc
Csf1r
Tmem119
r_P = 0.9987
R^2 = 0.9974

Fig. S5: RNA-seq demonstrates acute and prolonged neuroinflammation and neuronal damage in hippocampal tissue after sepsis

(**A**) Enrichment maps of hippocampal gene expression were generated for sham and PCI treated animals 3 days following sepsis induction. Red dots indicate upregulated and blue dots indicate downregulated gene sets. (**B**) Upregulated enrichment analysis of GO-BP terms and KEGG pathways comparing hippocampal tissue of sham vs PCI at day 10 ($n = 4$, $padj < 0.05$) following sepsis induction. (**C**) Correlation of RNA-seq and qPCR expression (log2 fold change) of selected genes (*C1qc*, *Tmem119*, *Csf1r*) at day 3 following sepsis induction used for transcriptome validation and analyzed by the Pearson test ($R^2 = 0.9974$, $p = 0.0326$).

Fig. S6

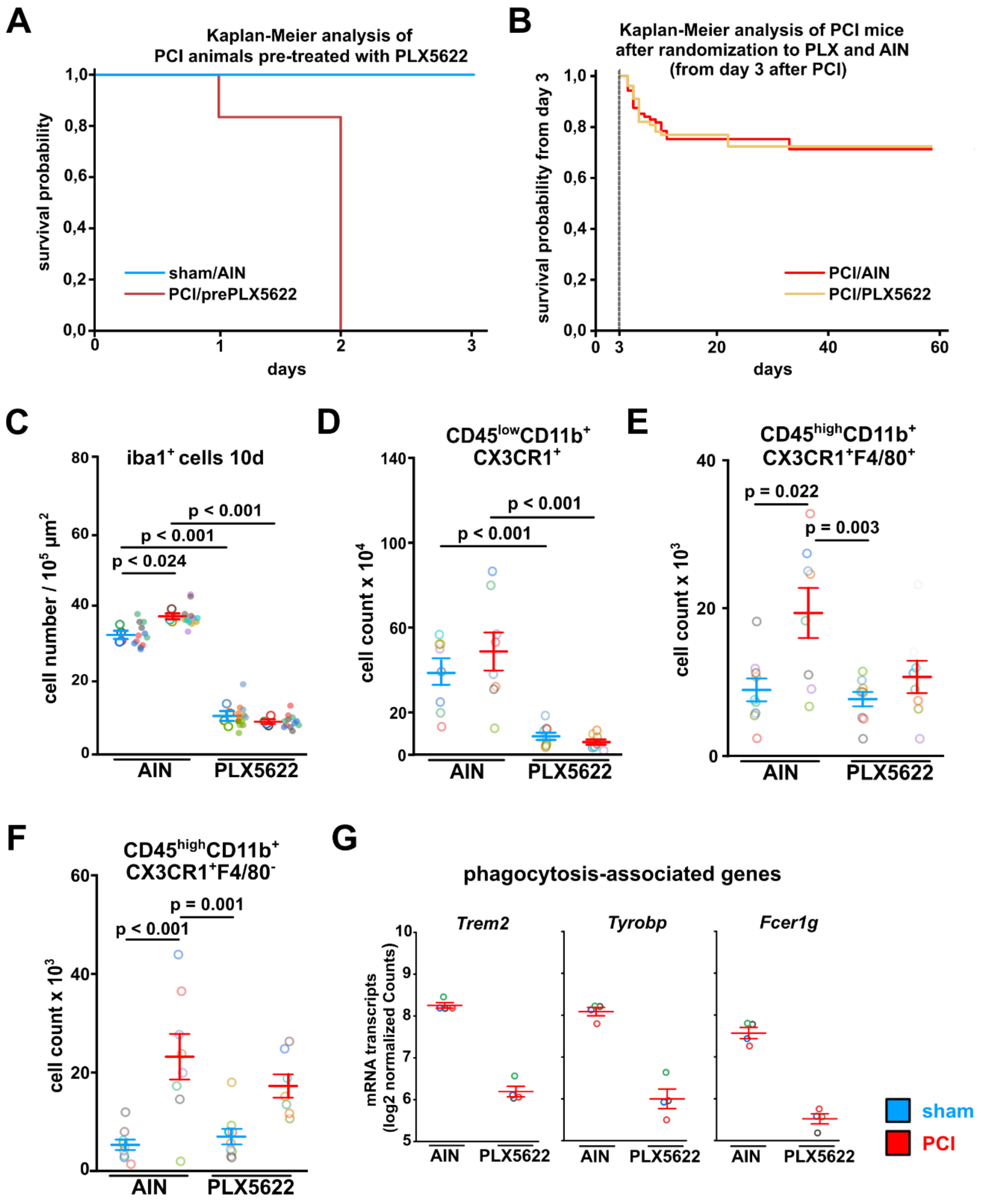
A
Kaplan-Meier analysis of
PCI animals pre-treated with PLX5622
survival probability
days
sham/AIN
PCI/prePLX5622
B
Kaplan-Meier analysis of PCI mice
after randomization to PLX and AIN
(from day 3 after PCI)
survival probability from day 3
days
PCI/AIN
PCI/PLX5622
C
iba1+ cells 10d
cell number / 10^5 µm^2
p < 0.024
p < 0.001
p < 0.001
AIN
PLX5622
D
CD45lowCD11b+
CX3CR1+
cell count x 10^4
p < 0.001
p < 0.001
AIN
PLX5622
E
CD45highCD11b+
CX3CR1+F4/80+
cell count x 10^3
p = 0.022
p = 0.003
AIN
PLX5622
F
CD45highCD11b+
CX3CR1+F4/80-
cell count x 10^3
p < 0.001
p = 0.001
AIN
PLX5622
G
phagocytosis-associated genes
Trem2
Tyrobp
Fcer1g
mRNA transcripts
(log2 normalized Counts)
AIN
PLX5622
sham
PCI

Fig. S6: CSF1-R inhibition differently affects brain resident and blood-derived mononuclear cells after sepsis

(**A**) Kaplan-Meier survival analysis of PCI mice treated 7 days with PLX5622 prior to sepsis induction and control mice (n=6/group) (Log-Rank-Test: p = 0,00121). (**B**) Kaplan-Meier survival analysis of PCI-AIN and PCI-PLX5622 treated animals after randomization to PLX5622 or AIN (from day 3 after PCI). Survival probability was normalized to surviving PCI mice on day 3 (Log-Rank-Test: p > 0.05). (**C**) Cell count of iba1 positive microglia in the hippocampal CA1 region of mice 10 days following sepsis induction with or without PLX5622 treatment (mice: n=4/group, images: n=3/mouse; One-way ANOVA with Bonferroni's multiple comparisons test). (**D**) Quantitative analysis microglia (CD45lowCD11b+CX3CR1+) using flow cytometry in whole brain tissue following sepsis induction with or without PLX5622 treatment (sham: n = 9; PCI: n = 8; sham-PLX5622: n = 9; PCI-PLX5622: n = 7; One-way ANOVA with Bonferroni's multiple comparisons test). (**E and F**) FACS analysis of $CD45^{high}CD11b^{+}CX3CR1^{+}F4/80^{+}$ macrophages and $CD45^{high}CD11b^{+}CX3CR1^{+}F4/80^{-}$ monocytes in whole brain lysate with or without PLX5622 treatment at day 10 following sepsis induction (sham: n = 9; PCI: n = 8; sham-PLX5622: n = 9; PCI-PLX5622: n = 7; One-way ANOVA with Bonferroni's multiple comparisons test). (**G**) Expression of DEGs encoding phagocytic proteins in PCI-AIN and PCI-PLX5622 treated animals at day 10 following sepsis induction (n = 4/group).

Data are presented as mean ± SEM.

Fig. S7

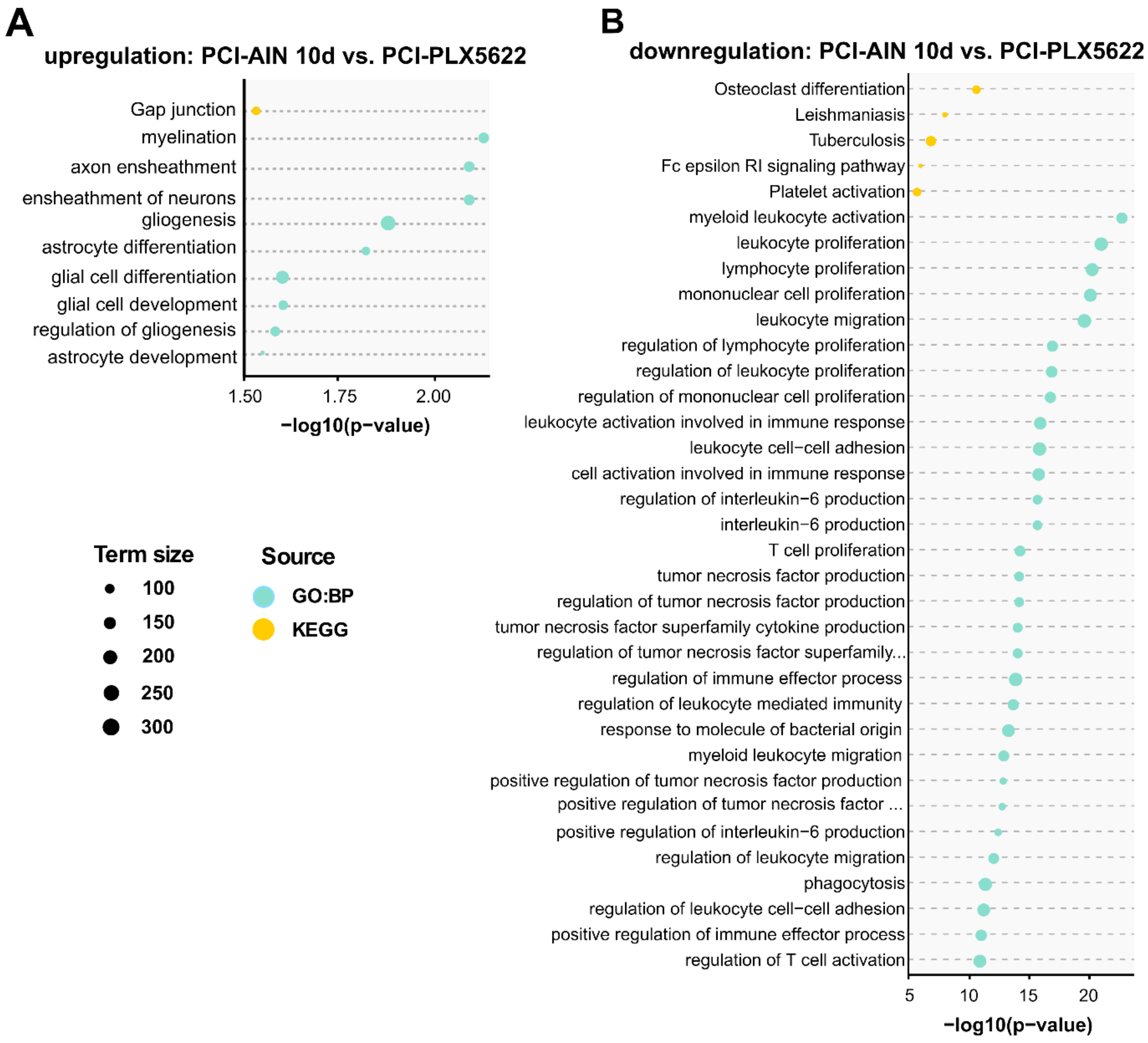

Fig. S7: CSF1-R inhibition reduces neuroinflammation

Upregulated **(A)** and downregulated **(B)** enrichment analysis of whole hippocampal tissue of GO-BP terms and KEGG pathways comparing the PCI-AIN vs PCI-PLX5622 group at day 10 (n = 4, padj < 0.05) following sepsis induction.

Fig. S8

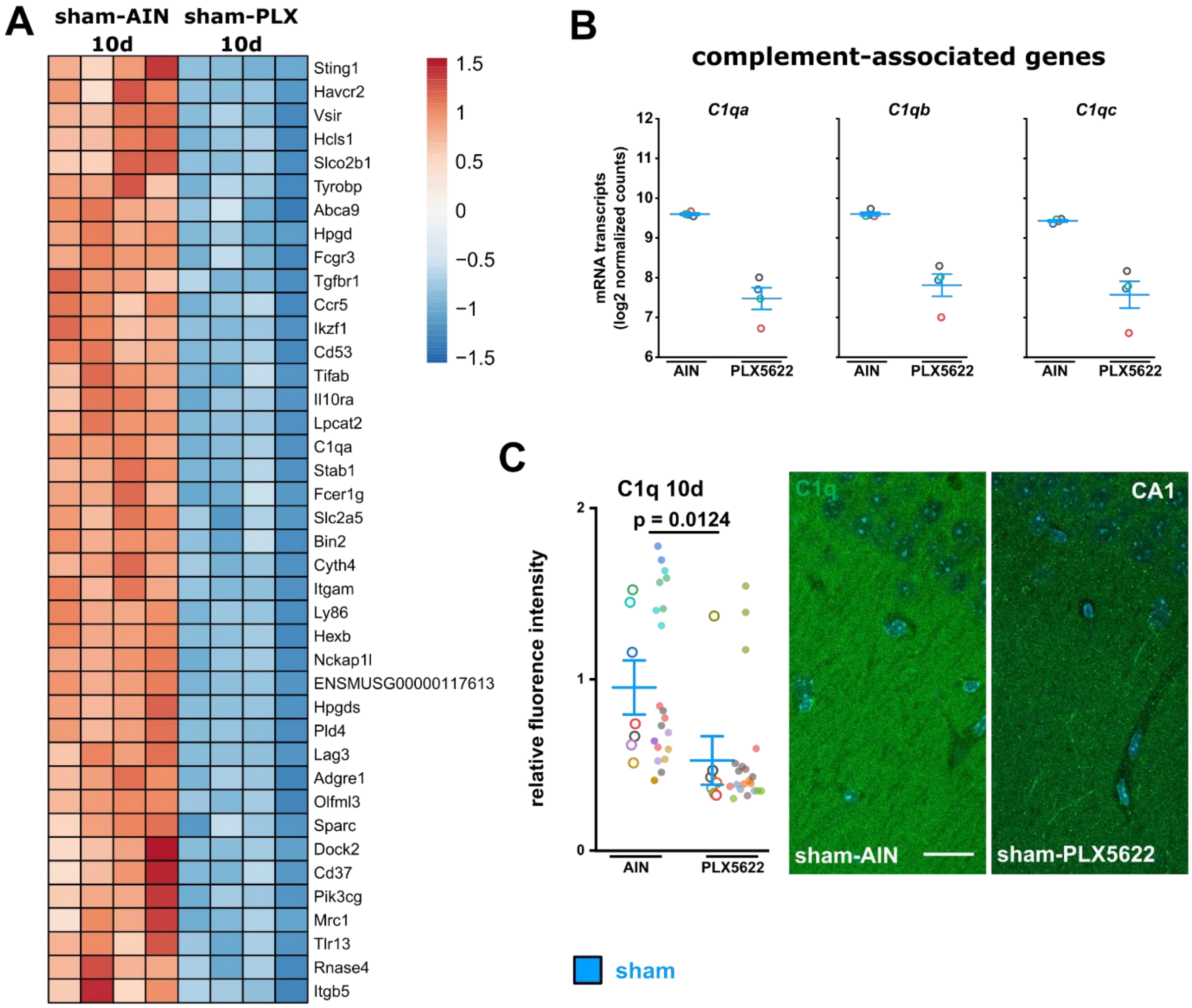

Fig. S8: Pharmacological microglia depletion downregulates C1q expression

(**A**) RNA-seq Expression heatmap of top 40 DEGs isolated from hippocampal tissue at day 10 (n = 4, padj < 0.05) in sham-AIN and sham-PLX5622 treated animals. The color scale represents the gene-wise z-score calculated from normalized gene expression levels. (**B**) Expression data of selected genes coding for complement factor C1q in sham-AIN and sham-PLX5622 treated animals at day 10 (n = 4/group). (**C**) Representative image (scale bar: 20µm) and quantification of C1q in sham-AIN and sham-PLX5622 animals at day 10 in the CA1 region of the hippocampus (sham-AIN: n = 7, sham-PLX5622: n = 7; images: n = 3/mouse; two-tailed Student's t-test). Data are presented as mean ± SEM.

Fig. S9

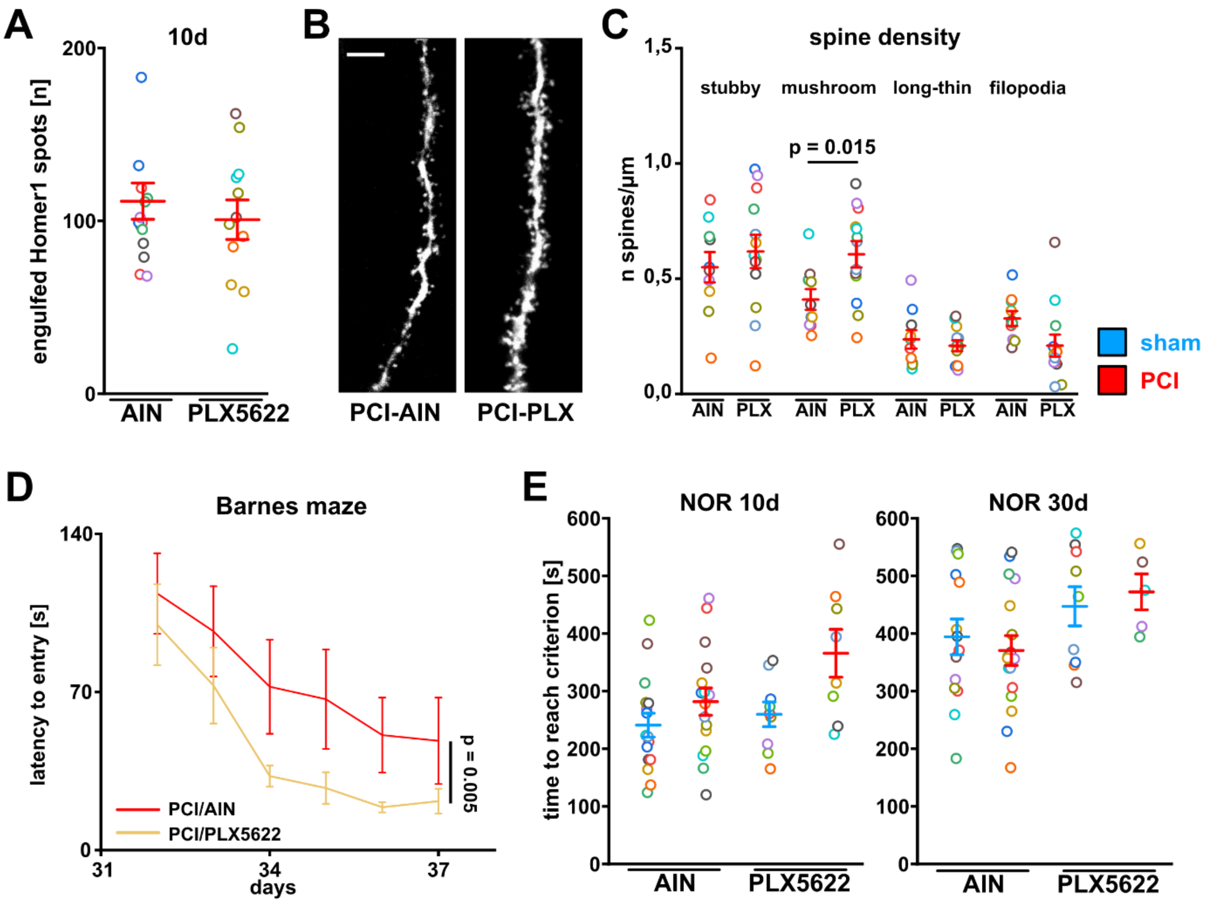
A
10d
engulfed Homer1 spots [n]
200
100
0
AIN
PLX5622
B
PCI-AIN
PCI-PLX
C
spine density
1,5
1,0
0,5
0,0
n spines/µm
stubby
mushroom
long-thin
filopodia
p = 0.015
AIN PLX AIN PLX AIN PLX AIN PLX
sham
PCI
D
Barnes maze
latency to entry [s]
140
70
0
31
34
37
days
PCI/AIN
PCI/PLX5622
p = 0.005
E
NOR 10d
NOR 30d
time to reach criterion [s]
600
500
400
300
200
100
0
AIN
PLX5622

Fig. S9: Pharmacological microglia depletion improves spine density and neurocognitive function

(**A**) Quantitative analysis of microglia engulfed Homer1 spots within microglia on day 10 following PCI induction in AIN (n = 13) or PLX5622 treated mice (n = 11; two-tailed Student's t-test). (**B-C**) Representative images of apical dendrites after sepsis (left: PCI-AIN; right: PCI-PLX5622; scale bar: 5µm). Quantification of apical synaptic spine density in dendrites of biocytin filled CA1 neurons following PCI in AIN (n = 10 cells from 3 mice) or PLX5622 treated mice (n = 13 cells from 4 mice; two-tailed Student's t-test). (**D**) Barnes maze test of PCI-AIN and PCI-PLX5622 treated animals from day 32 after PCI (n=11-12/group, p = 0.005; Two-way ANOVA repeated measurements with Bonferroni's multiple comparisons test.) (**E**) Locomotor activity measured by time to reach criterion (20 s at unknown and known objects) in NOR test at day 10 and 30 following sepsis induction.

Data are presented as mean ± SEM.

Table S6 Demographics and clinical data of post-mortem sepsis and control patients for histological analysis.

Table S1. Clinical data of post-mortem sepsis and control patients

case	cause of death at autopsy	underlying disease	age	gender
Sepsis				
1	septic MOF	mitral valve endocarditis	68	female
2	septic MOF	peritonitis	62	female
3	septic MOF	candida pneumonia	76	female
4	septic MOF	tricuspidal valve endocarditis	54	male
5	septic MOF	peritonitis	70	male
6	sepsis	pneumonia	76	male
7	septic MOF	small bowel perforation	69	male
8	septic MOF	acute infectious endocarditis	80	male
9	septic MOF	acute fungal endocarditis	54	male
controls				
10	MOF	portal vein thrombosis	72	female
11	cardiopulmonary failure	AMI	75	female
12	MOF	AMI	69	female
13	pulmonary embolism	deep vein thrombosis	58	male
14	shock	acute liver failure	70	male
15	cardiopulmonary failure	acute renal failure	70	male
16	AMI	type A dissection	74	male
17	respiratory failure	ARDS	79	male
18	cardiogenic shock	ventricular fibrillation	49	male

MOF = Multi-Organ Failure; ARDS = acute respiratory distress syndrome; AMI = acute myocardial infarction

Table S7 Demographics and clinical data of mild sepsis, severe sepsis and control patients for serum analysis.

Table 2. Clinical Data of Sepsis and Control Patients for serum analysis

**Case**	**SOFA-score**	**CRP**	**PCT**	**age**	**gender**
Control					
1	0	< 2,0		50	m
2	0	< 2,0		52	m
3	0	8,3		67	w
4	1	< 2,0		71	w
5	1	< 2,0		72	m
6	0	< 2,0		65	w
7	0	< 2,0		76	m
8	0	2,1		72	w
9	0	< 2,0		71	w
10	0	< 2,0		71	m
Mild Sepsis					
11	0	221,7	8,29	58	m
12	3	253,1	1,01	89	m
13	1	115,8	0,38	55	m
14	5	355,7	8,49	58	m
15	5	222,8	0,24	62	w
16	5	150,9	3,91	74	m
17	3	149,4	1,31	71	m
18	2	81,6	2,22	50	m
19	2	294	0,36	66	w
20	0	127,8	0,4	75	m
Severe Sepsis					
21	12	394,7	0,82	66	w
22	9	364,2	14,22	79	w
23	14	73,1	1,01	70	m
24	13	266,3	1,66	71	m
25	14	399	49,58	79	m
26	13	168	0,63	66	w
27	12	317	46,6	80	w
28	11	530,5	45,63	51	m
29	15	95,1	10,49	46	m
30	6	337,8	110,2	82	w

CRP = C-reactive protein; PCT = procalcitonin; SOFA = Sequential Organ Failure Assessment;

Supplementary data files

Data file S1: Differentially expressed genes from isolated microglia (day 3 and 20) and from hippocampus (day 3 and 10; AIN or PLX5622) after sepsis (Excel).

Data file S2: Intersection genes of RNA-seq data between isolated microglia at day 3 and 20 after sepsis (Excel).

Data file S3: Intersection genes of RNA-seq data between hippocampus tissue at day 3 and 10 after sepsis (Excel)

REFERENCES AND NOTES

1. C. Fleischmann, A. Scherag, N. K. Adhikari, C. S. Hartog, T. Tsaganos, P. Schlattmann, D. C. Angus, K. Reinhart, T. Assessment of global incidence and mortality of hospital-treated sepsis. Current estimates and limitations. *Am. J. Respir. Crit. Care Med.* **193**, 259–272 (2016).

2. H. Y. Chung, J. Wickel, F. M. Brunkhorst, C. Geis, Sepsis-associated encephalopathy: From delirium to dementia? *J. Clin. Med.* **9**, (2020).

3. R. Sonneville, E. de Montmollin, J. Poujade, M. Garrouste-Orgeas, B. Souweine, M. Darmon, E. Mariotte, L. Argaud, F. Barbier, D. Goldgran-Toledano, G. Marcotte, A. S. Dumenil, S. Jamali, G. Lacave, S. Ruckly, B. Mourvillier, J. F. Timsit, Potentially modifiable factors contributing to sepsis-associated encephalopathy. *Intensive Care Med.* **43**, 1075–1084 (2017).

4. T. E. Gofton, G. B. Young, Sepsis-associated encephalopathy. *Nat. Rev. Neurol.* **8**, 557–566 (2012).

5. A. Semmler, C. N. Widmann, T. Okulla, H. Urbach, M. Kaiser, G. Widman, F. Mormann, J. Weide, K. Fliessbach, A. Hoeft, F. Jessen, C. Putensen, M. T. Heneka, Persistent cognitive impairment, hippocampal atrophy and EEG changes in sepsis survivors. *J. Neurol. Neurosurg. Psychiatry* **84**, 62–69 (2013).

6. T. J. Iwashyna, E. W. Ely, D. M. Smith, K. M. Langa, Long-term cognitive impairment and functional disability among survivors of severe sepsis. *JAMA* **304**, 1787–1794 (2010).

7. D. Westhoff, J. Y. Engelen-Lee, I. C. M. Hoogland, E. M. A. Aronica, D. J. van Westerloo, D. van de Beek, W. A. van Gool, Systemic infection and microglia activation: A prospective postmortem study in sepsis patients. *Immun Ageing* **16**, 18 (2019).

8. M. Michels, B. Sonai, F. Dal-Pizzol, Polarization of microglia and its role in bacterial sepsis. *J. Neuroimmunol.* **303**, 90–98 (2017).

9. A. Trzeciak, Y. V. Lerman, T. H. Kim, M. R. Kim, N. Mai, M. W. Halterman, M. Kim, Long-term microgliosis driven by acute systemic inflammation. *J. Immunol.* **203**, 2979–2989 (2019).

10. R. M. Ransohoff, How neuroinflammation contributes to neurodegeneration. *Science* **353**, 777–783 (2016).

11. A. Nimmerjahn, F. Kirchhoff, F. Helmchen, Resting microglial cells are highly dynamic surveillants of brain parenchyma in vivo. *Science* **308**, 1314–1318 (2005).

12. C. N. Parkhurst, G. Yang, I. Ninan, J. N. Savas, J. R. Yates, III, J. J. Lafaille, B. L. Hempstead, D. R. Littman, W. B. Gan, Microglia promote learning-dependent synapse formation through brain-derived neurotrophic factor. *Cell* **155**, 1596–1609 (2013).

13. C. J. Bohlen, B. A. Friedman, B. Dejanovic, M. Sheng, Microglia in brain development, homeostasis, and neurodegeneration. *Annu. Rev. Genet.* **53**, 263–288 (2019).

14. M. W. Salter, B. Stevens, Microglia emerge as central players in brain disease. *Nat. Med.* **23**, 1018–1027 (2017).

15. R. C. Paolicelli, G. Bolasco, F. Pagani, L. Maggi, M. Scianni, P. Panzanelli, M. Giustetto, T. A. Ferreira, E. Guiducci, L. Dumas, D. Ragozzino, C. T. Gross, Synaptic pruning by microglia is necessary for normal brain development. *Science* **333**, 1456–1458 (2011).

16. A. Vukojicic, N. Delestree, E. V. Fletcher, J. G. Pagiazitis, S. Sankaranarayanan, T. A. Yednock, B. A. Barres, G. Z. Mentis, The classical complement pathway mediates microglia-dependent remodeling of spinal motor circuits during development and in SMA. *Cell Rep.* **29**, 3087–3100.e7 (2019).

17. C. Wang, H. Yue, Z. Hu, Y. Shen, J. Ma, J. Li, X. D. Wang, L. Wang, B. Sun, P. Shi, L. Wang, Y. Gu, Microglia mediate forgetting via complement-dependent synaptic elimination. *Science* **367**, 688–694 (2020).

18. B. Dejanovic, M. A. Huntley, A. De Maziere, W. J. Meilandt, T. Wu, K. Srinivasan, Z. Jiang, V. Gandham, B. A. Friedman, H. Ngu, O. Foreman, R. A. D. Carano, B. Chih, J. Klumperman, C. Bakalarski, J. E. Hanson, M. Sheng, Changes in the synaptic proteome in tauopathy and rescue of tau-induced synapse loss by C1q antibodies. *Neuron* **100**, 1322–1336.e7 (2018).

19. B. A. Gyorffy, J. Kun, G. Torok, E. Bulyaki, Z. Borhegyi, P. Gulyassy, V. Kis, P. Szocsics, A. Micsonai, J. Matko, L. Drahos, G. Juhasz, K. A. Kekesi, J. Kardos, Local apoptotic-like mechanisms underlie complement-mediated synaptic pruning. *Proc. Natl. Acad. Sci. U.S.A.* **115**, 6303–6308 (2018).

20. N. Scott-Hewitt, F. Perrucci, R. Morini, M. Erreni, M. Mahoney, A. Witkowska, A. Carey, E. Faggiani, L. T. Schuetz, S. Mason, M. Tamborini, M. Bizzotto, L. Passoni, F. Filipello, R. Jahn, B. Stevens, M. Matteoli, Local externalization of phosphatidylserine mediates developmental synaptic pruning by microglia. *EMBO J.* **39**, e105380 (2020).

21. M. R. Elmore, A. R. Najafi, M. A. Koike, N. N. Dagher, E. E. Spangenberg, R. A. Rice, M. Kitazawa, B. Matusow, H. Nguyen, B. L. West, K. N. Green, Colony-stimulating factor 1 receptor signaling is necessary for microglia viability, unmasking a microglia progenitor cell in the adult brain. *Neuron* **82**, 380–397 (2014).

22. E. Spangenberg, P. L. Severson, L. A. Hohsfield, J. Crapser, J. Zhang, E. A. Burton, Y. Zhang, W. Spevak, J. Lin, N. Y. Phan, G. Habets, A. Rymar, G. Tsang, J. Walters, M. Nespi, P. Singh, S. Broome, P. Ibrahim, C. Zhang, G. Bollag, B. L. West, K. N. Green, Sustained microglial depletion with CSF1R inhibitor impairs parenchymal plaque development in an Alzheimer's disease model. *Nat. Commun.* **10**, 3758 (2019).

23. R. J. Henry, R. M. Ritzel, J. P. Barrett, S. J. Doran, Y. Jiao, J. B. Leach, G. L. Szeto, J. Wu, B. A. Stoica, A. I. Faden, D. J. Loane, Microglial depletion with CSF1R inhibitor during chronic phase of experimental traumatic brain injury reduces neurodegeneration and neurological deficits. *J. Neurosci.* **40**, 2960–2974 (2020).

24. A. W. Lemstra, J. C. Groen in't Woud, J. J. Hoozemans, E. S. van Haastert, A. J. Rozemuller, P. Eikelenboom, W. A. van Gool, Microglia activation in sepsis: A case-control study. *J. Neuroinflammation* **4**, 4 (2007).

25. C. Kozlowski, R. M. Weimer, An automated method to quantify microglia morphology and application to monitor activation state longitudinally in vivo. *PLOS ONE* **7**, e31814 (2012).

26. S. E. Hickman, N. D. Kingery, T. K. Ohsumi, M. L. Borowsky, L. C. Wang, T. K. Means, J. E. Khoury, The microglial sensome revealed by direct RNA sequencing. *Nat. Neurosci.* **16**, 1896–1905 (2013).

27. C. Chhatbar, M. Prinz, The roles of microglia in viral encephalitis: From sensome to therapeutic targeting. *Cell. Mol. Immunol.* **18**, 250–258 (2021).

28. A. Shemer, I. Scheyltjens, G. R. Frumer, J. S. Kim, J. Grozovski, S. Ayanaw, B. Dassa, H. Van Hove, L. Chappell-Maor, S. Boura-Halfon, D. Leshkowitz, W. Mueller, N. Maggio, K. Movahedi, S. Jung, Interleukin-10 prevents pathological microglia hyperactivation following peripheral endotoxin challenge. *Immunity* **53**, 1033–1049.e7 (2020).

29. C. Sousa, A. Golebiewska, S. K. Poovathingal, T. Kaoma, Y. Pires-Afonso, S. Martina, D. Coowar, F. Azuaje, A. Skupin, R. Balling, K. Biber, S. P. Niclou, A. Michelucci, Single-cell transcriptomics reveals distinct inflammation-induced microglia signatures. *EMBO Rep.* **19**, e46171 (2018).

30. F. A. Gonnert, P. Recknagel, M. Seidel, N. Jbeily, K. Dahlke, C. L. Bockmeyer, J. Winning, W. Losche, R. A. Claus, M. Bauer, Characteristics of clinical sepsis reflected in a reliable and reproducible rodent sepsis model. *J. Surg. Res.* **170**, e123–134 (2011).

31. L. Gaetani, K. Blennow, P. Calabresi, M. Di Filippo, L. Parnetti, H. Zetterberg, Neurofilament light chain as a biomarker in neurological disorders. *J. Neurol. Neurosurg. Psychiatry* **90**, 870–881 (2019).

32. J. Ehler, A. Petzold, M. Wittstock, S. Kolbaske, M. Gloger, J. Henschel, A. Heslegrave, H. Zetterberg, M. P. Lunn, P. S. Rommer, A. Grossmann, T. Sharshar, G. Richter, G. Noldge-Schomburg, M. Sauer, The prognostic value of neurofilament levels in patients with sepsis-associated encephalopathy—A prospective, pilot observational study. *PLOS ONE* **14**, e0211184 (2019).

33. M. I. Fonseca, S. H. Chu, M. X. Hernandez, M. J. Fang, L. Modarresi, P. Selvan, G. R. MacGregor, A. J. Tenner, Cell-specific deletion of C1qa identifies microglia as the dominant source of C1q in mouse brain. *J. Neuroinflammation* **14**, 48 (2017).

34. S. S. Holden, F. C. Grandi, O. Aboubakr, B. Higashikubo, F. S. Cho, A. H. Chang, A. O. Forero, A. R. Morningstar, V. Mathur, L. J. Kuhn, P. Suri, S. Sankaranarayanan, Y. Andrews-Zwilling, A. J. Tenner,

A. Luthi, E. Aronica, M. R. Corces, T. Yednock, J. T. Paz, Complement factor C1q mediates sleep spindle loss and epileptic spikes after mild brain injury. *Science* **373**, eabj2685 (2021).

35. D. T. Skelly, E. W. Griffin, C. L. Murray, S. Harney, C. O'Boyle, E. Hennessy, M. A. Dansereau, A. Nazmi, L. Tortorelli, J. N. Rawlins, D. M. Bannerman, C. Cunningham, Acute transient cognitive dysfunction and acute brain injury induced by systemic inflammation occur by dissociable IL-1-dependent mechanisms. *Mol. Psychiatry* **24**, 1533–1548 (2019).

36. D. Tejera, D. Mercan, J. M. Sanchez-Caro, M. Hanan, D. Greenberg, H. Soreq, E. Latz, D. Golenbock, M. T. Heneka, Systemic inflammation impairs microglial Aβ clearance through NLRP3 inflammasome. *EMBO J.* **38**, e101064 (2019).

37. T. Manabe, I. Racz, S. Schwartz, L. Oberle, F. Santarelli, J. V. Emmrich, J. J. Neher, M. T. Heneka, Systemic inflammation induced the delayed reduction of excitatory synapses in the CA3 during ageing. *J. Neurochem.* **159**, 525–542 (2021).

38. I. Michailidou, J. G. Willems, E. J. Kooi, C. van Eden, S. M. Gold, J. J. Geurts, F. Baas, I. Huitinga, V. Ramaglia, Complement C1q-C3-associated synaptic changes in multiple sclerosis hippocampus. *Ann. Neurol.* **77**, 1007–1026 (2015).

39. M. I. Fonseca, C. H. Kawas, J. C. Troncoso, A. J. Tenner, Neuronal localization of C1q in preclinical Alzheimer's disease. *Neurobiol. Dis.* **15**, 40–46 (2004).

40. S. Hong, V. F. Beja-Glasser, B. M. Nfonoyim, A. Frouin, S. Li, S. Ramakrishnan, K. M. Merry, Q. Shi, A. Rosenthal, B. A. Barres, C. A. Lemere, D. J. Selkoe, B. Stevens, Complement and microglia mediate early synapse loss in Alzheimer mouse models. *Science* **352**, 712–716 (2016).

41. C. M. Comim, T. Barichello, D. Grandgirard, F. Dal-Pizzol, J. Quevedo, S. L. Leib, Caspase-3 mediates in part hippocampal apoptosis in sepsis. *Mol. Neurobiol.* **47**, 394–398 (2013).

42. T. Manabe, M. T. Heneka, Cerebral dysfunctions caused by sepsis during ageing. *Nat. Rev. Immunol.* **22**, 444–458 (2022).

43. L. G. Bodea, Y. Wang, B. Linnartz-Gerlach, J. Kopatz, L. Sinkkonen, R. Musgrove, T. Kaoma, A. Muller, L. Vallar, D. A. Di Monte, R. Balling, H. Neumann, Neurodegeneration by activation of the microglial complement-phagosome pathway. *J. Neurosci.* **34**, 8546–8556 (2014).

44. T. Zrzavy, R. Hoftberger, T. Berger, H. Rauschka, O. Butovsky, H. Weiner, H. Lassmann, Pro-inflammatory activation of microglia in the brain of patients with sepsis. *Neuropathol. Appl. Neurobiol.* **45**, 278–290 (2019).

45. F. Lei, N. Cui, C. Zhou, J. Chodosh, D. G. Vavvas, E. I. Paschalis, CSF1R inhibition by a small-molecule inhibitor is not microglia specific; affecting hematopoiesis and the function of macrophages. *Proc. Natl. Acad. Sci. U.S.A.* **117**, 23336–23338 (2020).

46. K. N. Green, J. D. Crapser, L. A. Hohsfield, To kill a microglia: A case for CSF1R inhibitors. *Trends Immunol.* **41**, 771–784 (2020).

47. M. Kitic, P. See, J. Bruttger, F. Ginhoux, A. Waisman, Novel microglia depletion systems: A genetic approach utilizing conditional diphtheria toxin receptor expression and a pharmacological model based on the blocking of macrophage colony-stimulating factor 1 receptor. *Methods Mol. Biol.* **2034**, 217–230 (2019).

48. A. Bedolla, A. Taranov, F. Luo, J. Wang, F. Turcato, E. M. Fugate, N. H. Greig, D. M. Lindquist, S. A. Crone, J. Goto, Y. Luo, Diphtheria toxin induced but not CSF1R inhibitor mediated microglia ablation model leads to the loss of CSF/ventricular spaces in vivo that is independent of cytokine upregulation. *J. Neuroinflammation* **19**, 3 (2022).

49. A. Trzeciak, R. K. Mongre, M. R. Kim, K. Lim, R. A. Madero, C. N. Parkhurst, A. P. Pietropaoli, M. Kim, Neutrophil heterogeneity in complement C1q expression associated with sepsis mortality. *Front. Immunol.* **13**, 965305 (2022).

50. M. Absinta, D. Maric, M. Gharagozloo, T. Garton, M. D. Smith, J. Jin, K. C. Fitzgerald, A. Song, P. Liu, J. P. Lin, T. Wu, K. R. Johnson, D. B. McGavern, D. P. Schafer, P. A. Calabresi, D. S. Reich, A lymphocyte-microglia-astrocyte axis in chronic active multiple sclerosis. *Nature* **597**, 709–714 (2021).

51. F. Weber, B. Bohrmann, J. Niewoehner, J. A. A. Fischer, P. Rueger, G. Tiefenthaler, J. Moelleken, A. Bujotzek, K. Brady, T. Singer, M. Ebeling, A. Iglesias, P. O. Freskgard, Brain shuttle antibody for Alzheimer's disease with attenuated peripheral effector function due to an inverted binding mode. *Cell Rep.* **22**, 149–162 (2018).

52. S. M. Coldewey, C. Neu, P. Baumbach, A. Scherag, B. Goebel, K. Ludewig, F. Bloos, M. Bauer, Identification of cardiovascular and molecular prognostic factors for the medium-term and long-term outcomes of sepsis (ICROS): Protocol for a prospective monocentric cohort study. *BMJ Open* **10**, e036527 (2020).

53. N. Percie du Sert, V. Hurst, A. Ahluwalia, S. Alam, M. T. Avey, M. Baker, W. J. Browne, A. Clark, I. C. Cuthill, U. Dirnagl, M. Emerson, P. Garner, S. T. Holgate, D. W. Howells, N. A. Karp, S. E. Lazic, K. Lidster, C. J. MacCallum, M. Macleod, E. J. Pearl, O. H. Petersen, F. Rawle, P. Reynolds, K. Rooney, E. S. Sena, S. D. Silberberg, T. Steckler, H. Wurbel, The ARRIVE guidelines 2.0: Updated guidelines for reporting animal research. *PLoS Biol.* **18**, e3000410 (2020).

54. B. Grünewald, J. Wickel, N. Hahn, F. Hörhold, H. Rupp, H.-Y. Chung, H. Haselmann, A. S. Strauss, L. Schmidl, N. Hempel, L. Grünewald, A. Urbach, M. Bauer, K. V. Toyka, M. Blaess, R. A. Claus, R. König, C. Geis, Targeted rescue of synaptic plasticity improves cognitive decline after severe systemic inflammation. *bioRxiv* 2021.03.04.433352 [**Preprint**] (2021).

55. M. Steinwand, P. Droste, A. Frenzel, M. Hust, S. Dubel, T. Schirrmann, The influence of antibody fragment format on phage display based affinity maturation of IgG. *MAbs* **6**, 204–218 (2014).

56. E. Arduin, S. Arora, P. R. Bamert, T. Kuiper, S. Popp, S. Geisse, R. Grau, T. Calzascia, G. Zenke, J. Kovarik, Highly reduced binding to high and low affinity mouse Fc gamma receptors by L234A/L235A and N297A Fc mutations engineered into mouse IgG2a. *Mol. Immunol.* **63**, 456–463 (2015).

57. K. T. Schneider, T. Kirmann, E. V. Wenzel, J. H. Grosch, S. Polten, D. Meier, M. Becker, P. Matejtschuk, M. Hust, G. Russo, S. Dubel, Shelf-life extension of Fc-fused single chain fragment variable antibodies by lyophilization. *Front. Cell. Infect. Microbiol.* **11**, 717689 (2021).

58. F. Bertoglio, V. Fuhner, M. Ruschig, P. A. Heine, L. Abassi, T. Klunemann, U. Rand, D. Meier, N. Langreder, S. Steinke, R. Ballmann, K. T. Schneider, K. D. R. Roth, P. Kuhn, P. Riese, D. Schackermann, J. Korn, A. Koch, M. Z. Chaudhry, K. Eschke, Y. Kim, S. Zock-Emmenthal, M. Becker, M. Scholz, G. Moreira, E. V. Wenzel, G. Russo, H. S. P. Garritsen, S. Casu, A. Gerstner, G. Roth, J. Adler, J. Trimpert, A. Hermann, T. Schirrmann, S. Dubel, A. Frenzel, J. Van den Heuvel, L. Cicin-Sain, M. Schubert, M. Hust, A SARS-CoV-2 neutralizing antibody selected from COVID-19 patients binds to the ACE2-RBD interface and is tolerant to most known RBD mutations. *Cell Rep.* **36**, 109433 (2021).

59. A. Dobin, C. A. Davis, F. Schlesinger, J. Drenkow, C. Zaleski, S. Jha, P. Batut, M. Chaisson, T. R. Gingeras, STAR: Ultrafast universal RNA-seq aligner. *Bioinformatics* **29**, 15–21 (2013).

60. Y. Liao, G. K. Smyth, W. Shi, featureCounts: An efficient general purpose program for assigning sequence reads to genomic features. *Bioinformatics* **30**, 923–930 (2014).

61. M. I. Love, W. Huber, S. Anders, Moderated estimation of fold change and dispersion for RNA-seq data with DESeq2. *Genome Biol.* **15**, 550 (2014).

62. L. Kolberg, U. Raudvere, I. Kuzmin, J. Vilo, H. Peterson, gprofiler2—An R package for gene list functional enrichment analysis and namespace conversion toolset g:Profiler. *F1000Res* **9**, 709 (2020).

63. R. Edgar, M. Domrachev, A. E. Lash, Gene expression omnibus: NCBI gene expression and hybridization array data repository. *Nucleic Acids Res.* **30**, 207–210 (2002).

64. M. Leger, A. Quiedeville, V. Bouet, B. Haelewyn, M. Boulouard, P. Schumann-Bard, T. Freret, Object recognition test in mice. *Nat. Protoc.* **8**, 2531–2537 (2013).

65. D. R. Bentley, S. Balasubramanian, H. P. Swerdlow, G. P. Smith, J. Milton, C. G. Brown, K. P. Hall, D. J. Evers, C. L. Barnes, H. R. Bignell, J. M. Boutell, J. Bryant, R. J. Carter, R. Keira Cheetham, A. J. Cox, D. J. Ellis, M. R. Flatbush, N. A. Gormley, S. J. Humphray, L. J. Irving, M. S. Karbelashvili, S. M. Kirk, H. Li, X. Liu, K. S. Maisinger, L. J. Murray, B. Obradovic, T. Ost, M. L. Parkinson, M. R. Pratt, I. M. Rasolonjatovo, M. T. Reed, R. Rigatti, C. Rodighiero, M. T. Ross, A. Sabot, S. V. Sankar, A. Scally, G. P. Schroth, M. E. Smith, V. P. Smith, A. Spiridou, P. E. Torrance, S. S. Tzonev, E. H. Vermaas, K. Walter, X. Wu, L. Zhang, M. D. Alam, C. Anastasi, I. C. Aniebo, D. M. Bailey, I. R. Bancarz, S.

Banerjee, S. G. Barbour, P. A. Baybayan, V. A. Benoit, K. F. Benson, C. Bevis, P. J. Black, A. Boodhun, J. S. Brennan, J. A. Bridgham, R. C. Brown, A. A. Brown, D. H. Buermann, A. A. Bundu, J. C. Burrows, N. P. Carter, N. Castillo, E. C. M. Chiara, S. Chang, R. Neil Cooley, N. R. Crake, O. O. Dada, K. D. Diakoumakos, B. Dominguez-Fernandez, D. J. Earnshaw, U. C. Egbujor, D. W. Elmore, S. S. Etchin, M. R. Ewan, M. Fedurco, L. J. Fraser, K. V. Fuentes Fajardo, W. Scott Furey, D. George, K. J. Gietzen, C. P. Goddard, G. S. Golda, P. A. Granieri, D. E. Green, D. L. Gustafson, N. F. Hansen, K. Harnish, C. D. Haudenschild, N. I. Heyer, M. M. Hims, J. T. Ho, A. M. Horgan, K. Hoschler, S. Hurwitz, D. V. Ivanov, M. Q. Johnson, T. James, T. A. Huw Jones, G. D. Kang, T. H. Kerelska, A. D. Kersey, I. Khrebtukova, A. P. Kindwall, Z. Kingsbury, P. I. Kokko-Gonzales, A. Kumar, M. A. Laurent, C. T. Lawley, S. E. Lee, X. Lee, A. K. Liao, J. A. Loch, M. Lok, S. Luo, R. M. Mammen, J. W. Martin, P. G. McCauley, P. McNitt, P. Mehta, K. W. Moon, J. W. Mullens, T. Newington, Z. Ning, B. Ling Ng, S. M. Novo, M. J. O'Neill, M. A. Osborne, A. Osnowski, O. Ostadan, L. L. Paraschos, L. Pickering, A. C. Pike, A. C. Pike, D. Chris Pinkard, D. P. Pliskin, J. Podhasky, V. J. Quijano, C. Raczy, V. H. Rae, S. R. Rawlings, A. Chiva Rodriguez, P. M. Roe, J. Rogers, M. C. Rogert Bacigalupo, N. Romanov, A. Romieu, R. K. Roth, N. J. Rourke, S. T. Ruediger, E. Rusman, R. M. Sanches-Kuiper, M. R. Schenker, J. M. Seoane, R. J. Shaw, M. K. Shiver, S. W. Short, N. L. Sizto, J. P. Sluis, M. A. Smith, J. Ernest Sohna Sohna, E. J. Spence, K. Stevens, N. Sutton, L. Szajkowski, C. L. Tregidgo, G. Turcatti, S. Vandevondele, Y. Verhovsky, S. M. Virk, S. Wakelin, G. C. Walcott, J. Wang, G. J. Worsley, J. Yan, L. Yau, M. Zuerlein, J. Rogers, J. C. Mullikin, M. E. Hurles, N. J. McCooke, J. S. West, F. L. Oaks, P. L. Lundberg, D. Klenerman, R. Durbin, A. J. Smith, Accurate whole human genome sequencing using reversible terminator chemistry. *Nature* **456**, 53–59 (2008).

66. K. J. Livak, T. D. Schmittgen, Analysis of relative gene expression data using real-time quantitative PCR and the $2^{-\Delta\Delta C}{}_{T}$ method. *Methods* **25**, 402–408 (2001).

67. P. Shannon, A. Markiel, O. Ozier, N. S. Baliga, J. T. Wang, D. Ramage, N. Amin, B. Schwikowski, T. Ideker, Cytoscape: A software environment for integrated models of biomolecular interaction networks. *Genome Res.* **13**, 2498–2504 (2003).

68. D. Merico, R. Isserlin, O. Stueker, A. Emili, G. D. Bader, Enrichment map: A network-based method for gene-set enrichment visualization and interpretation. *PLOS ONE* **5**, e13984 (2010).

69. M. Kucera, R. Isserlin, A. Arkhangorodsky, G. D. Bader, AutoAnnotate: A cytoscape app for summarizing networks with semantic annotations. *F1000Res* **5**, 1717 (2016).

70. T. Gruter, F. E. Mollers, A. Tietz, J. Dargvainiene, N. Melzer, A. Heidbreder, C. Strippel, A. Kraft, R. Hoftberger, F. Schoberl, F. S. Thaler, J. Wickel, H. Y. Chung, F. Seifert, M. Tschernatsch, M. Nagel, J. Lewerenz, S. Jarius, B. C. Wildemann, L. de Azevedo, F. Heidenreich, R. Heusgen, U. Hofstadt-van Oy, A. Linsa, J. J. Maass, T. Menge, M. Ringelstein, D. J. Pedrosa, J. Schill, T. Seifert-Held, C. Seitz, S. Tonner, C. Urbanek, S. Zittel, R. Markewitz, M. Korporal-Kuhnke, T. Schmitter, C. Finke, N. Bruggemann, C. I. Bien, I. Kleiter, R. Gold, K. P. Wandinger, G. Kuhlenbaumer, F. Leypoldt, I. Ayzenberg, E. Clinical, serological and genetic predictors of response to immunotherapy in anti-IgLON5 disease. *Brain* **146**, 600–611 (2023).

71. C. A. Butler, A. S. Popescu, E. J. A. Kitchener, D. H. Allendorf, M. Puigdellivol, G. C. Brown, Microglial phagocytosis of neurons in neurodegeneration, and its regulation. *J. Neurochem.* **158**, 621–639 (2021).

72. H. Haselmann, L. Ropke, C. Werner, A. Kunze, C. Geis, Interactions of human autoantibodies with hippocampal GABAergic synaptic transmission-analyzing antibody-induced effects ex vivo. *Front. Neurol.* **6**, 136 (2015).
